# Additions to the checklist of Scoliidae, Sphecidae, Pompilidae and Vespidae of Peru, with notes on the endemic status of some species (Hymenoptera, Aculeata)

**DOI:** 10.3897/zookeys.519.6501

**Published:** 2015-08-31

**Authors:** Eduardo Fernando dos Santos, Yuri Campanholo Grandinete, Fernando Barbosa Noll

**Affiliations:** 1 Departamento de Zoologia e Botânica, Instituto de Biociências, Letras e Ciências Exatas, Universidade Estadual Paulista “Júlio de Mesquita Filho”. Rua Cristóvão Colombo, 2265, Jd. Nazareth, 15054-000, São José do Rio Preto, SP, Brazil; 2 Departamento de Biologia, Faculdade de Filosofia, Ciências e Letras de Ribeirão Preto, Universidade de São Paulo. Av. Bandeirantes, 3900, 14040-901, Ribeirão Preto, SP, Brazil

**Keywords:** Apoidea, Vespoidea, endemic species, social wasps, potter wasps, spider wasps, digger wasps

## Abstract

The first checklist of the Peruvian Hymenoptera listed 1169 species and subspecies of aculeate wasps, including 173 species of Pompilidae, seven of Scoliidae, 39 of Sphecidae and 403 of Vespidae. Herein are reported 32 species as new for Peru based mainly on the collection of the Natural History Museum, London. The loss of the endemic status of two species is also reported: *Entypus peruvianus* (Rohwer) (Pompilidae: Pepsinae) and *Omicron ruficolle schunkei* Giordani Soika (Vespidae: Eumeninae).

## Introduction

Pompilidae, Scoliidae and Vespidae together with other seven families have been included in Vespoidea, while Sphecidae has been positioned in Apoidea, both included in Hymenoptera Aculeata (Brothers and Finnamore 1993, Hanson and Gauld 2006). Rasmussen and Asenjo (2009) compiled the first checklist of Hymenoptera Aculeata for Peru, including 1158 species and subspecies, with the majority (52%) of them belonging to Pompilidae, Scoliidae, Sphecidae and Vespidae. In addition, it included 226 endemic species for Peru, with 50 Pompilidae, one Scoliidae, two Sphecidae and 67 Vespidae.

Pompilidae includes approximately 5,000 species worldwide, with 850 neotropical species among 56 genera (Fernández 2000), of which 159 species in 33 genera are recorded for Peru (Rasmussen and Asenjo 2009). Scoliidae is a small component of the aculeate hymenopteran fauna, with nearly 300 species in the world, 47 of which are found in the New World (Hanson 2006). For Peru, Rasmussen and Asenjo (2009) listed eight species in three subgenera of *Campsomeris* Guerin. The biology of Scoliidae is poorly known, and the existing studies indicate that species are idiobiont ectoparasitoids of coleopteran larvae (Brothers and Finnamore 1993, Hanson 2006, Fernández 2006).

Sphecidae and Vespidae comprise, respectively, more than 660 and 5,000 described species of predatory wasps throughout the world (Goulet and Huber 1993, Pickett and Carpenter 2010). Rasmussen and Asenjo (2009) listed 39 species of 12 genera of Sphecidae classified in four subfamilies: Ammophilinae, Chloriontinae, Sceliphrinae and Sphecinae, all of them with neotropical representatives (Goulet and Huber 1993, Hanson and Menke 2006). Species of Ammophilinae are predators of Lepidoptera or Symphyta (Hymenoptera) larvae, while species of Chloriontinae have been recorded as predators of Gryllidae (Orthoptera) and Blattaria, Sceliphrinae as predator of Araneae and Blattaria, and Sphecinae as predators of Orthoptera (Goulet and Huber 1993, Hanson and Menke 2006). Vespidae, in turn, are classified in six subfamilies (Carpenter 1982, Pickett and Carpenter 2010), three of them, Eumeninae, Masarinae and Polistinae, known from the neotropical region (Carpenter 1993, Carpenter et al. 2006, Hermes et al. 2013). Rasmussen and Asenjo (2009) recorded 403 species and subspecies of 52 genera of Vespidae for Peru. Masarinae are solitary wasps and commonly known as “pollen wasps”, since their larvae are fed with pollen and nectar (Gess 1996) while Eumeninae and Polistinae, respectively, are solitary or social wasps preying on lepidopteran larvae as food for their larvae (Bohart and Stange 1965, West-Eberhard et al 2006).

Contributing to knowledge of the neotropical hymenopteran fauna, herein are presented new species records of Pompilidae, Scoliidae, Sphecidae and Vespidae from Peru.

## Material and methods

The checklist of Rasmussen and Asenjo (2009) is updated, including new species records from Peru based on specimens deposited mainly in the Natural History Museum, London (NHM, Dr. David Norton (curator of the Apoidea collection) and Dr. Gavin Broad (curator of the Vespoidea collection)), with some sporadic records from other institutions, as follows: American Museum of Natural History (AMNH, Dr. James M. Carpenter), Instituto de Biociências, Letras e Ciências Exatas da Universidade Estadual de São Paulo (IBILCE-UNESP, Dr. Fernando Barbosa Noll), Instituto Fundación Miguel Lillo (IFML, Emilia Constanza Pérez), Museu Paraense Emílio Goeldi (MPEG, Dr. Orlando Tobias Silveira), Museo di Storia Naturale di Venezia (MSNVE, Dr. Marco Uliana) and Museu de Zoologia da Universidade de São Paulo (MZUSP, Dr. Carlos Roberto Ferreira Brandão), Smithsonian Institution National Museum of Natural History (USMN, Dr. Sean Brady). Species identification of Pompilidae and Sphecidae deposited in the NHM and included herein were confirmed. Dubious identification were not included in the present list. For Scoliidae and Vespidae, only specimens labeled with specialist’s identification were included. In this way, only species of Scoliidae determined by the Dr. James Chester Bradly (1884–1975) or Dr. Johan George Betrem (1899-1980) were considered. These two entomologists were the two main specialists in Scoliidae. All identification deposited in AMNH, IBILCE-UNESP, IFML, MPEG, MSNVE, MZUSP and USMN was confirmed. In addtition,

The present checklist is organized alphabetically, following Rasmussen and Asenjo (2009) and adopting the most traditional family classification, in that Pompilidae, Scoliidae and Vespidae are placed in Vespoidea and Sphecidae in Apoidea with three subfamilies, differing from Rasmussen and Asenjo (2009) who adopted four subfamilies for Sphecidae. For Pompilidae, Rasmussen and Ansejo (2009) used the Pitts et al.’ (2008) proposal, however, they adopted a hypothesis among 720 other equally parsimonious ones. Then, we decided to use the classification proposed by Shimizu (1994), which positioned *Epipompilus* in Epipompilinae and *Notocyphus* in Notocyphinae. The Vespidae tribe classification was based on Hermes et al. (2014).

All new records are assigned a cross (†). Abbreviations for the departments of Peru used in the present checklist are the same as in Rasmussen and Asenjo (2009), as follows: AM, Amazonas; AN, Ancash; AP, Apurímac; AR, Arequipa; CA, Cajamarca; CL, Callao; CU, Cusco; HU, Huánuco; HV, Huancavelica; JU, Junín; LA, Lambayeque; LI, Lima; LL, La Libertad; LO, Loreto; MD, Madre de Dios; PA, Pasco; PI, Piura; SM, San Martin; UC, Ucayali.

## Results and discussion

In total, 32 species are recorded for the first time from Peru, 20 species of Pompilidae, three of Scoliidae, five of Sphecidae and four of Vespidae, and more than 250 records are new for Peruvian departments or lacalities. Moreover, seven species of Vespidae have been described by Cooper (2010, 2013a, 2013b, 2014), Silveira (2013) and Santos et al. (2015) for Peru, after the publication of Rasmussen and Asenjo’s (2009) checklist. Santos et al. (2015) also resurrected *Protopolybia diligens* (Smith). Based on the taxonomic update and the present compilation, 1196 species of Hymenoptera Aculeata are now known for Peru (see Suppl. material 2: Updated checklist). Pompilidae is currently represented by 173 species belonging to 33 genera, Scoliidae by eleven species in two genera, Sphecidae by 44 species of 12 genera and Vespidae by 405 species and sub-species of 53 genera. In the case of Scoliidae, a species of Scoliinae, Scolia (Hesperoscolia) rufiventris (Fabricius), is recorded for the first time for Peru.

In addition, the NHM’s collection includes specimens of *Entypus peruvianus* (Rohwer) from Argentina and Bolivia, indicating that it is not a Peruvian endemic species. Another taxon that has lost its endemic status is *Omicron ruficolle schunkei* Giordani Soika, which has been recorded in Brazil in this work.

### *Entypus peruvianus* (Rohwer, 1913)

**Specimen data.** ARGENTINA: 1♀, S. de Estero, 37-47k. S.e. Anatuya, 20.xi.1979, C. & M. Vardy [NHM]. 1♀ and 1♂, Salta, Orán, Abra Grande, x.18–25 1968, C. Porter [NHM]. 1♀ and 1♂, Salta, Orán, Abra Grande, x.18–25 1968, C. Porter [NHM]. BOLIVIA: 1♀, La Paz, Zongo Valley, Cahua, 1400m, 22–23.vi.1979, M. Cooper, B.M. 1979-397 [NHM]. 3♀♀, La Paz, Coroico, 20.v.1989, 1200m, M. Cooper, 1979-216 [NHM]. 1♀, La Paz, Chulumani, 1700m, 25.iii.1979, M. Cooper, B.M. 1979-216 [NHM].

### *Omicron ruficolle schunkei* Giordani Soika, 1978

**Specimen data.** BRAZIL: 5♀♀, SP, Pindorama, 21°13'27"S 48°55'35"W, 08/viii/2014, Soleman, R.A. col. [IBILCE-UNESP]

Futhermore, Castro-Huertas et al. (2014) recorded other three species of Pompilidae from Colombia (Pepsinae: *Ageniella pretiosa* Banks and *Mystacagenia bellula* Evans; Pompilinae: Aporus (Aporus) cuzco Evans), and Santos et al. (2015) another one of Polistinae (Vespidae) from Brazil (*Protopolybia chanchamayensis* Bequaert) that were regarded as endemic by Rasmussen and Asenjo (2009). On the other hand, Silveira (2013) and Santos et al. (2015) described new species of Polistinae (*Mischocyttarus tayacaja* Silveira; *Protopolybia similis* Santos, Silveira & Carpenter), and Cooper (2013a) described one of Eumeninae (*Pararhaphidoglossa carpenteri* Cooper) that are known only from Peru. These aspects indicate the hymenopteran fauna of several South American regions are still poorly sampled, and new collecting efforts and identification efforts of already collected material should increase the number of species recorded for Peru.

### SPHECIDAE

**Chloriontinae**

***Chlorion* Latreille, 1802**

*viridicoeruleum* Lepeletier & Seville, 1828†: UC (Pucallpa)

**Sceliphrinae**

***Penepodium* Menke in Bohart & Menke, 1976**

*gorianum* (Lepeletier, 1845): UC (Pucallpa)

*romandinum* (de Saussure, 1867): UC (Pucallpa)

***Podium* Fabricius, 1804 [Kohl 1902]**

*agile* Kohl, 1902†: UC (Pucallpa)

*denticulatum* Smith, 1856: UC (Pucallpa)

*kohlii* Zavattari, 1908: UC (Pucallpa)

*rufipes* Fabricius, 1804†: UC (Pucallpa)

**Sphecinae**

***Sphex* Linnaeus, 1758**

Subgenus: *Sphex* Linnaeus, 1758

*nitidiventris* Spinola, 1853†: UC (Pucallpa)

*tinctipennis* Cameron, 1888†: CU (Atalaya), HU (Previsto), UC (B. Abad)

### POMPILIDAE

**Ceropalinae**

***Ceropales* Latreille, 1796**

Subgenus: *Bifidoceropales* Priesner, 1969

isolde subsp. isolde (Banks, 1945)†: LL (Trujillo)

***Irenangelus* Schulz, 1906**

*reversus* (Smith, 1873): UC (B. Abad)

**Epipompilinae**

***Epipompilus* Kohl, 1884**

*williamsi* (Banks, 1947)†: PA (Oxapampa)

**Pepsinae**

**Ageniellini**

***Ageniella* Banks, 1912**

Subgenus: *Alasagenia* Banks, 1944

*fortipes* (Smith, 1873) †: UC (Pucallpa)

*pilifrons* (Cameron, 1912) †: HU (Monzón-Rondos River), JU (Chanchamayo), UC (B. Abad, Pucallpa)

Subgenus: *Ageniella* Banks, 1912

*ruficeps* (Smith, 1864) †: UC (Pucallpa)

***Auplopus* Spinola, 1841**

*deceptor* (Smith, 1873) †: HU (Cord. Azul)

***Priocnemella* Banks, 1925**

hexagona subsp. omissa Banks, 1946: HU (Tingo María), JU (Chanchamayo), UC (Atalaya, Pucallpa)

**Pepsini**

***Caliadurgus* Pate, 1946**

*pretiosus* (Fox, 1897)†: PA (Oxapampa)

***Entypus* Dahlbom, 1843**

*decoloratus* (Lepeletier, 1845)†: HU (Monzón)

*dumosus* (Spinola, 1851)†: LA (Jayanca), LI (Matucana), LL (Simbal)

*gigas* (Fabricius, 1804)†: UC (Pucallpa)

*molestus* (Banks, 1946): Endemic, LA (Lambayeque), LI (Matucana), LL (Simbal)

*nitidus* (Banks, 1946): HU (Cayumba)

*peruvianus* (Rohwer, 1913): CU (Apurimac River, Machu Picchu, Urubamba River)

**Pompilinae**

**Aporini**

***Aporus* Spinola, 1808**

Subgenus: *Aporus* Spinola, 1808

*cuzco* Evans, 1973: CU (Urubamba River)

Subgenus: *Neoplaniceps* Bradley, 1944

*umbratilis* Evans, 1966: CU (Urubamba), HU (Tingo María)

Subgenus: *Notoplaniceps* Bradley, 1944

*canescens* Smith, 1873: MD (Pantiacolla)

***Euplaniceps* Haupt, 1930**

*varia* Bradley, 1944: CU (Atalaya), UC (B. Abad)

**Pompilini**

***Anoplius* Dufour, 1834**

Subgenus: *Anopliodes* Banks, 1939

*varius* (Fabricius, 1804): HU (Cord. Azul, Tingo María), UC (B. Abad)

Subgenus: *Anoplius* Dufour, 1834

*ambatoensis* (Cameron, 1903)†: CU (Urubamba), LI (Chosica)

Subgenus: *Arachnophroctonus* Howard, 1901

*inculcatrix* (Cameron, 1912): JU (Chanchamayo), LO (Est. Jenaro Herrera)

***Austrochares* Banks, 1947**

*elsinore* Banks, 1947: Endemic, CL (Ventanilla), LA (Chiclayo, Jayanca, Lambayeque, Olmos), LL (Simbal, Trujillo)

***Balboana* Banks, 1944**

*auripennis* (Fabricius, 1804): CU (Quincemil), HU (Tingo María), UC (Atalaya, Pucallpa)

*manifestata* (Smith, 1864) †: LO (Est. Jenaro Herrera)

***Evagetes* Lepeletier, 1845**

*copiosus* Banks, 1947†: CU (Cusco)

***Paracyphononyx* Gribodo, 1884**

*unicolor* (Smith, 1879) †: CU (Apurimac River, Machu Picchu, Urubamba River), HU (Tingo María), JU (Chanchamayo)

***Poecilopompilus* Howard, 1901**

*rubricatus* (Smith, 1879): LA (Chiclayo, Jayanca, Lambayeque), LI (Cieneguilla, Lima), LL (Trujillo, Jequetepeque River)

***Priochilus* Banks, 1944**

*captivum* (Fabricius, 1804)†: LO (Contamana, Iquitos), PA (Pichis), UC (Pucallpa)

*formosus* Banks, 1944: CU (Quincemil), JU (Chanchamayo), SM (Nuevo Progreso)

gloriosum subsp. gloriosum (Cresson, 1869)†: JU (Chanchamayo), UC (B. Abad, Pucallpa)

*gracillimus* (Smith, 1855) (= *scrupulum* (Fox, 1897)): HU (Tingo María), UC (Pucallpa)

*pectoralis* (Smith, 1855) (= *imperius* Banks, 1944): CA (Jaén), LO (Est. Jenaro Herrera), MD (Atalaya), UC (Pucallpa).

regius subsp. regius (Fabricius, 1804): HU (Cayumba, Tingo María, Yanayacu), JU (Chanchamayo), UC (Atalaya, B. Abad, Pucallpa)

*ruficoxalis* (Fox, 1897)†: UC (Pucallpa)

*sericeifrons* (Fox, 1897): CU (Quincemil), HU (Monzón- Rondos River, Tingo María), UC (B. Abad, Pucallpa).

splendidulum subsp. splendidulum (Fabricius, 1804): HU (Cord. Azul, Tingo María), LO (Est. Jenaro Herrera), UC (B. Abad, Pucallpa).

*veraepascis* (Cameron, 1893): LO (Est. Jenaro Herrera).

***Tachypompilus* Ashmead, 1902**

*pallidus* Banks, 1947: Endemic, LI (Chosica), LL (Simbal)

**Notocyphinae**

***Notocyphus* Smith, 1855**

*crassicornis* Smith, 1864†: JU (Chanchamayo), LO (Est. Jenaro Herrera), UC (Pucallpa).

*laetabilis* (Smith, 1873)†: UC (Pucallpa).

*multipicta* Smith, 1873†: LO (Est. Jenaro Herrera), UC (B. Abad, Pucallpa)

*saevissimus* Smith, 1855†: UC (Pucallpa)

*thetis* Banks, 1945†: CU (Atalaya), UC (Pucallpa)

### SCOLIIDAE

**Campsomerinae**

***Campsomeris* Guérin-Méneville, 1839**

Subgenus: *Aelocampsomeris* Bradley, 1957

*variegata* Fabricius, 1793: JU (Chanchamayo)

Subgenus: *Lissocampsomeris* Bradley, 1957

*arneohirta* (Fox, 1896)†: JU (Chanchamayo), UC (B. Abad)

*wesmaeli* (Lepeletier, 1845)†: CU (Atalaya), HU (Monzón – Rondos River), JU (Chanchamayo), UC (B. Abad, Pucallpa)

**Scoliinae**

***Scolia* Fabricius, 1775**

Subgenus: *Hesperoscolia* Bradley, 1974

*rufiventris* (Fabricius, 1804)†: JU (Chanchamayo), UC (B. Abad, Pucallpa).

### VESPIDAE

**Eumeninae**

**Eumenini**

***Brachymenes* Giordani Soika, 1961**

*wagnerianus* (de Saussure, 1875): UC (Cord. Azul).

***Eumenes* Latreille, 1802**

Subgenus: *Zeteumenoides* Giordani Soika, 1972

*filiformis* (de Saussure, 1855): MD

***Omicron* de Saussure, 1855**

*paranymphum* (Zavattari, 1912): UC (Previsto)

***Pachymenes* de Saussure, 1852**

*consuetus* Giordani Soika, 1990: MD, JU (Chanchamayo) (= obscurus subsp. consuetus Giordani Soika, 1990 – Grandinete et al. 2014)

*ghilianii* Spinola, 1851: MD (= ghilianii subsp. olivaceus (de Saussure, 1875); =*peruanus* Schrottky, 1911 – see Grandinete et al. 2014)

*orellanae* (Schulz, 1905): HU (Tingo María), LO (Iquitos), MD (Res. Nac. Tambopata), UC (B. Abad) (= orellanae subsp. orellanae (Schulz, 1905), = orellanae subsp. vardyi Giordani Soika, 1990 – see Grandinete et al. 2014)

***Pararhaphidoglossa* von Schulthess, 1910**

*carpenteri* Cooper, 2013: Endemic, LO (Iquitos) [Cooper 2013a]

*gribodoi* (Zavattari, 1912): JU (Pan de Azucar) [Cooper 2013b]

*mestiza* Giordani Soika, 1978: HU (Yanayacu) [Cooper 2012]

*schunkei* Cooper, 2014: UC (B. Abad) [Cooper 2014]

*sulcata* Cooper, 2013: LO (Iquitos) [Cooper 2013b]

***Pirhosigma* Giordani Soika, 1978**

mearimense subsp. mearimense (Zavattati, 1912)†: UC (Pucallpa)

***Sphaeromenes* Giordani Soika, 1978**

*discrepatus* Giordani Soika, 1978: LI (Santa Rosa de Quives), LL (Trujillo)

**Odynerini**

***Hypalastoroides* de Saussure, 1852**

Subgenus: *Hypalastoroides* de Saussure, 1852

*brasiliensis* (de Saussure, 1856): LO (Iquitos)

***Hypodynerus* de Saussure, 1855**

*arequipensis* (du Buysson, 1913): JU (Yanamaria)

*obscuripennis* (Spinola, 1851): AR (Arequipa)

***Pseudodynerus* de Saussure, 1855**

*subapicalis* (Fox, 1902): HU (Huánuco)

**Zethini**

***Zethus* Fabricius, 1804**

Subgenus: *Zethus* Fabricius, 1804

*cataractae* Cooper, 2010: JU (Quebrada Mala Noche) [Cooper 2010]

**Polistinae**

**Epiponini**

***Agelaia* Lepeletier de Saint Fargeau, 1836**

*myrmecophila* (Ducke, 1905): HU (Llullapichis), LO

***Brachygastra* Perty, 1833**

*albula* Richards, 1978†: CU (Cadena, Cusco). [Andena and Carpenter 2012]

*augusti* (de Saussure, 1854): CU (Cadena), MD (Res. Nac. Tambopata), LO (Estiron), UC (Cord. Azul, Pucallpa)

*baccalaurea* (R. von Ihering, 1903): CU (Machu Picchu), PI (Huacabamba)

*bilineolata* Spinola, 1841: CU (Cadena), LO (Dos de Mayo, Iquitos), PA (Cerro de Pasco)

*lecheguana* (Latreille, 1824): CA (Quebrada Nancho), LA (Jequetepeque), LL (Pascamayo), UC (Yarinacocha)

*propodealis* Bequaert, 1943: CU (Cadena), MD (Puerto Maldonado)

*scutellaris* (Fabricius, 1804): HU (Llullapichis River)

*smithii* (de Saussure, 1854): LA (Jequetepeque), LL (Pascamayo), LO (Galicia)

***Chartergellus* Bequaert, 1938**

*fulvus* Fox, 1898: HU (Tingo María), LO (Mishuyacu)

***Polybia* Lepeletier, 1836**

Subgenus: *Alpha* de Saussure, 1854

*bifasciata* de Saussure, 1854: JU (Chanchamayo), UC (B. Abad)

*quadricincta* de Saussure, 1854: UC (Pucallpa)

Subgenus: *Apopolybia* Richards, 1978

*jurinei* de Saussure, 1854: LO (Iquitos), UC (Previsto, Pucallpa)

Subgenus: *Cylindroeca* Richards, 1978

*dimidiata* (Olivier, 1791): JU (Chanchamayo), UC (Previsto, Pucallpa)

Subgenus: *Formicicola* Richards, 1978

*rejecta* (Fabricius, 1798): HU (Yanayacu), UC (Previsto, Pucallpa)

Subgenus: *Myrapetra* White, 1841

*bistriata* (Fabricius, 1804): UC (Pucallpa)

*catillifex* Möbius, 1856: UC (Atalaya, B. Abad, Previsto)

*diguetana* du Buysson, 1905: PA (Oxapampa), UC (Pucallpa)

*fastidiosuscula* de Saussure, 1854: JU (Chanchamayo), LA (Chiclayo)

*parvulina* Richards, 1970†: JU (Chanchamayo), UC (Previsto)

Subgenus: *Platypolybia* Richards, 1978

*incerta* Ducke, 1907: HU (Tingo María), JU (Chanchamayo), UC (San Alejandro)

procellosa subsp. dubitata Ducke, 1910: UC (B. Abad, Previsto)

Subgenus: *Polybia* Lepeletier de Saint Fargeau, 1836

*striata* (Fabricius, 1787): HU (Tingo María), UC (Pucallpa)

Subgenus: *Trichinothorax* Carpenter & Day, 1988

*micans* Ducke, 1904: PA (Puerto Bermúdez)

rufitarsis subsp. peruviana Bequaert, 1943: LO (Iquitos), UC (B. Abad)

*sericea* (Olivier, 1791): CA (Jaén)

tinctipennis subsp. tinctipennis Fox, 1898: UC (Pucallpa)

*velutina* Ducke, 1905: UC (Atalaya, Boq. Abad, Previsto), HU (Tingo María)

***Protopolybia* Ducke, 1905**

*acutiscutis* (Cameron, 1907): LO (Nanay River)

*bella* (R. von Ihering, 1903): CU (Paucartambo), HU (Tingo María)

chartergoides subsp. chartergoides (Gribodo, 1891): UC (Pucallpa)

*clypeata* Santos, Silveira & Carpenter (2015): LO (Galícia, San Pedro, Yarin) [Santos et al. 2015]

*diligens* (Smith, 1857): LO (Napo) [Santos et al. 2015]

*sedula* (de Saussure, 1854): UC (Pucallpa)

*similis* Santos, Silveira & Carpenter, 2015: JU (San Ramón) [Santos et al. 2015]

**Mischocyttarini**

***Mischocyttarus* de Saussure, 1853**

Subgenus: *Phi* de Saussure, 1854

*alfkenii* Ducke, 1904: LO [Silveira, 2013]

*flavicornis* Zikán, 1949 (=flavicornis subsp. nigricornis Zikán, 1949 – see Silveira 2013): CU (Cusco, Urubamba), JU (Satipo) [Silveira 2013]

*flavoniger* Zikán, 1949: JU (Chanchamayo) [Silveira 2013]

*tayacaja* Silveira, 2013: HV (Campo Armiño) [Silveira 2013]

**Polistini**

***Polistes* Latreille, 1802**

Subgenus: *Aphanilopterus* Meunier, 1888

*claripennis* Ducke, 1904: UC (Pucallpa)

*deceptor* Schulz, 1905: JU (Chanchamayo), SM (Rioja), UC (Pucallpa)

*maranonensis* Willink, 1964: AM (Utcubamba)

pacificus subsp. modestus Smith, 1868†: HU (Tingo María)

*testaceicolor* Bequaert, 1937: UC (Previsto, Pucallpa)

## Appendix I

**Table:** Localities reported in the checklist including coordinates and altitude range (m a.s.l.).

Department	Locality	Longitude	Latitude	Altitude
**AMAZONAS**	Utcubamba, Valley	-78.55250	-5.53500	1700
**AREQUIPA**	Arequipa	-71.51667	-16.38333	2300
**CAJAMARCA**	Quebrada Nancho	-79.25000	-6.96667	400
	Jaén	-78.81667	-5.70000	750
**CALLAO**	Ventanilla	-77.07361	-11.85556	97
**CUSCO**	Apurimac River (68 km west Cusco)	-72.11667	-13.64667	2400
	Atalaya	-71.35000	-12.90000	600
	Cadena	-70.717	-13.4000	1600
	Cusco	-71.98333	-13.51667	3400–4200
	Machu Picchu	-72.54579	-13.16319	2385–2700
	Paucartambo	-71.60000	-13.31667	2900
	Quincemil	-70.76667	-13.23333	630–650
	Urubamba	-72.28530	-13.23250	3500
	Urubamba River	-72.11667	-13.30417	2900
**HUANUCO**	Cayumba	-75.95000	-9.50000	2000
	Cord. Azul (= Cordillera Azul)	-75.93333	-9.15000	1600
	Llullapichis	-74.93333	-9.61667	260
	Monzón, Valley	-76.40000	-9.28333	950
	Monzón, Valley – Rondos River	-76.08333	-9.25000	950
	Tingo María	-76.00000	-9.30000	670
	Yanayacu	-74.97611	-9.55722	200
**HUANCAVELICA**	Campo Armiño	-74.65000	-12.35000	1600–1880
**JUNÍN**	Chanchamayo, Valley of	-75.31667	-11.05000	700–1300
	Pan de Azucar (Km 83 Tarma to San Ramon)	-75.45250	-11.17830	1400
	Quebrada Mala Noche	-75.41110	-11.12690	1300–1500
	San Ramón	-75.35000	-11.13333	900
**JUNÍN**	Satipo	-74.63674	-11.25423	631
	Yanamaria [Ana Maria?], near Jauja	-75.66410	-11.79200	3470
**LAMBAYEQUE**	Chiclayo	-79.85000	-6.76667	21
	Jayanca	-79.82194	-6.39083	61
	Lambayeque	-79.90000	-6.70000	20
	Olmos	-79.75000	-5.98333	150
**LIMA**	Chosica	-76.70000	-11.93333	850
	Cieneguilla	-76.81160	-12.11730	550
	Lima	-77.03333	-12.05000	50
	Matucana	-76.38550	-11.84576	2400
	Santa Rosa de Quives	-76.78877	-11.66904	1100–1550
**LA LIBERTAD**	Jequetepeque River	-78.87861	-7.50496	818
	Pascamayo	-79.56667	-7.41667	20
	Simbal	-78.81667	-7.96667	576
	Trujillo	-79.03333	-8.11667	20
**LORETO**	Contamana	-75.00000	-7.35000	150
	Dos de Mayo	-75.11700	-6.40000	100–124
	Estación Jenaro Herrera	-73.65011	-4.89861	121
	Estirón, Ampiyacu River	-72.00800	-3.36800	93
	Galicia	-73.80000	-5.28333	700
	Iquitos	-73.25000	-3.75000	100
	Mishuyacu, River	-73.30000	-3.78333	120
	Nanay River	-73.38889	-3.81806	97
	Napo – Sucusari River	-72.91850	-3.26650	84
	San Pedro	-74.25000	-4.45000	75–170
	Yarin, Quebrada	-74.03333	-3.90000	127
**MADRE DE DIOS**	Avispas	-70.35000	-12.98333	350–400
	Atalaya, Manu Nat. Park	-71.72139	-11.85639	305–1000
	Puerto Maldonado	-69.18333	-12.60000	200
	Reserva Nacional de Tambopata	-69.28300	-12.85000	236
	Pantiacolla - Upper Madre de Dios River	-71.23180	-12.65570	404
**PASCO**	Cerro de Pasco	-76.26200	-10.68600	4310
	Oxapampa	-75.40000	-10.58333	1800
**PASCO**	Pichis, River	-74.93333	-9.90000	300
	Puerto Bermúdez	-74.93333	-10.30000	3–700
**PIURA**	Huancabamba	-79.44300	-5.25700	2713
**SAN MARTIN**	Nuevo Progreso	-76.32465	-8.448889	511
	Rioja	-77.16667	-6.6667	900
**UCAYALI**	Atalaya	-73.76667	-10.73333	250
	B. Abad (= Boquerón del Padre Abad)	-75.68061	-9.07082	500
	Previsto - Cord. Azul (= Cordillera Azul)	-75.83200	-9.07500	700–2000
	Previsto	-75.63333	-9.05000	420–500
	Pucallpa	-74.53055	-8.37971	180–500
	San Alejandro	-75.76667	-9.08333	1300–1600
	Yarinacohca	-74.60000	-8.35000	150–300

## Supplementary materials

Supplementary material 1 Full Checklist of the Peruvian Scoliidae, Sphecidae, Pompilidae and Vespidae Data type: occurence This dataset is made available under the Open Database License (http://opendatacommons.org/licenses/odbl/1.0/). The Open Database License (ODbL) is a license agreement intended to allow users to freely share, modify, and use this Dataset while maintaining this same freedom for others, provided that the original source and author(s) are credited.Eduardo Fernando dos Santos, Yuri Campanholo Grandinete, Fernando Barbosa Noll

Supplementary material 2 Additions to the checklist of Scoliidae, Sphecidae, Pompilidae and Vespidae of Peru, with notes on the endemic status of some species (Hymenoptera, Aculeata) Data type: Checklist This dataset is made available under the Open Database License (http://opendatacommons.org/licenses/odbl/1.0/). The Open Database License (ODbL) is a license agreement intended to allow users to freely share, modify, and use this Dataset while maintaining this same freedom for others, provided that the original source and author(s) are credited.Eduardo Fernando dos Santos, Yuri Campanholo Grandinete, Fernando Barbosa Noll
