# Supplementary material for: Additions to the checklist of Scoliidae, Sphecidae, Pompilidae and Vespidae of Peru, with notes on the endemic status of some species (Hymenoptera, Aculeata)
Source: Zookeys. 2015 Aug 31;(519):33–48. doi: 10.3897/zookeys.519.6501 (PMC4591603; doi:10.3897/zookeys.519.6501)
Supplement: Supplementary material 1 — Full Checklist of the Peruvian Scoliidae, Sphecidae, Pompilidae and Vespidae [file zookeys-519-033-s001.docx]

**Additions to the checklist of Scoliidae, Sphecidae, Pompilidae and Vespidae of Peru, with notes on the endemic status of some species (Hymenoptera: Aculeata)**

Eduardo Fernando dos Santos^1^, Yuri Campanholo Grandinete^1,2^, Fernando Barbosa Noll^1^

1. Departamento de Zoologia e Botânica, Instituto de Biociências, Letras e Ciências Exatas, Universidade Estadual Paulista “Júlio de Mesquita Filho”. Rua Cristóvão Colombo, 2265, Jd. Nazareth, 15054-000, São José do Rio Preto, SP, Brazil.
2. Departamento de Biologia, Faculdade de Filosofia, Ciências e Letras de Ribeirão Preto, Universidade de São Paulo. Av. Bandeirantes, 3900, 14040-901, Ribeirão Preto, SP, Brazil.

*Correspondence email: efs.wasp@gmail.com

**Full Checklist of the Peruvian Scoliidae, Sphecidae, Pompilidae and Vespidae**

All new records are assigned a cross (†). Abbreviations for the departments of Peru used in the present checklist are the same as in Rasmussen and Asenjo (2009), as follows: AM, Amazonas; AN, Ancash; AP, Apurímac; AR, Arequipa; AY, Ayacucho; CA, Cajamarca; CL, Callao; CU, Cusco; HU, Huánuco; HV, Huancavelica; IC, Ica; JU, Junín; LA, Lambayeque; LI, Lima; LL, La Libertad; LO, Loreto; MD, Madre de Dios; MO, Moquegua; PA, Pasco; PI, Piura; PU, Puno; SM, San Martin; TA, Tacna; TU, Tumbes; UC, Ucayali.

**SPHECIDAE**

**Ammophilinae**

***Ammophila* Kirby, 1798**

*laeviceps* Smith, 1873

*lampei* Strand, 1910: CU, PU

*rufipes* Guérin-Méneville, 1831: LA (Lambayeque), LI (Callao, Cañete, Chosica, Lima), LL (Trujillo)

***Eremnophila* Menke, 1964**

*opulenta* (Guérin-Méneville, 1838): MD (Pakitza)

**Chloriontinae**

***Chlorion* Latreille, 1802**

*mirandum* (Kohl, 1890): AM (Santiago), UC (Ucayali)

*viridicoeruleum* Lepeletier & Seville, 1828†: UC (Pucallpa)

**Sceliphrinae**

***Chalybion* Dahlbom, 1843**

*californicum* (de Saussure, 1867): LI

***Dynatus* Lepeletier, 1845**

*nigripes* subsp. *nigripes* (Westwood, 1832): JU

***Penepodium* Menke in Bohart & Menke, 1976**

*gorianum* (Lepeletier, 1845)

*haematogastrum* (Spinola, 1851)

*mocsaryi* (Kohl, 1902)

*princeps* (Kohl, 1902)

*romandinum* (de Saussure, 1867)

***Podium* Fabricius, 1804 [Kohl 1902]**

*agile* Kohl, 1902†: UC (Pucallpa)

*denticulatum* Smith, 1856: UC† (Pucallpa)

*kohlii* Zavattari, 1908: UC† (Pucallpa)

*plesiosaurus* (Smith, 1873): MD (Puerto Maldonado)

*rufipes* Fabricius, 1804†: UC (Pucallpa)

***Sceliphron* Klug, 1801**

Subgenus: *Sceliphron* Klug, 1801

*asiaticum* (Linnaeus, 1758)

*caementarium* (Drury, 1773): LI, PI (Sullana)

*fistularium* (Dahlbom, 1843): JU (Satipo), LI, LO (Yurimaguas), PI (Sullana), UC (Pucallpa)

***Trigonopsis* Perty, 1833**

*cooperi* Vardy, 1978: HU (Tingo María)

*menkei* Vardy, 1978: LO (Iquitos)

*neotropica* Vardy, 1978: HU (Tingo María)

*richardsi* Vardy, 1978: JU (Satipo), SM (Moyobamba), UC (Pucallpa)

*rufiventris* (Fabricius, 1804): HU (Cayumba, Pachitea), JU (Chanchamayo, El Campamento, Satipo), LO (Iquitos, San Roque, Tapiche, Yurimaguas), MD (Pakitza), UC (Atalaya, B. Abad, Pucallpa)

*schunkei* Vardy, 1978: LO (San Roque), UC (Pucallpa)

*succinea* Vardy, 1978: UC (Pucallpa)

*violascens* (Dalla Torre, 1897): AM (Santiago), CU (Cosñipata), HU (Pachitea, Previsto, Tingo María), JU (Chanchamayo), MD (Pakitza)

**Sphecinae**

***Isodontia* Patton, 1880**

*costipennis* (Spinola, 1851): (=cyanipennis Fabricius, 1793)

*dolosa* (Kohl, 1895)

***Prionyx* Vander Linden, 1827**

*chilensis* (Spinola, 1851).

*erythrogastra* (Rohwer, 1913): CU (Cusco).

*herrerai* (Brèthes, 1926): Endemic, CU (Cusco).

*neoxenus* (Kohl, 1890): AR (Arequipa), LI (La Molina), PU.

*pseudostriatus* (Giner Marí, 1944): Endemic, LI.

*thomae* (Fabricius, 1775): LI

***Sphex* Linnaeus, 1758**

Subgenus: *Sphex* Linnaeus, 1758

*caliginosus* Erichson, 1849

*dorsalis* Lepeletier, 1845: AP, AY, HU

*ichneumoneus* (Linnaeus, 1758): LI (Lima)

*latro* Erichson, 1849

*nitidiventris* Spinola, 1853†: UC (Pucallpa)

*peruanus* Kohl, 1890: AN, AR (Arequipa), LI (Lima), PU

*tinctipennis* Cameron, 1888†: CU (Atalaya), HU (Previsto), UC (B. Abad).

**POMPILIDAE**

**Ceropalinae**

***Ceropales* Latreille, 1796**

Subgenus: *Bifidoceropales* Priesner, 1969

*abdominalis* Taschenberg, 1869: LO, UC (Pucallpa)

*cubensis* subsp. *cooperi* Móczár, 1990: LL (Simbal)

*cubensis* subsp. *vardyi* Móczár, 1990: LA (Lambayeque), LL (Moche, Trujillo)

*luctuosa* subsp. *luctuosa* Smith, 1864: PA (Oxapampa)

*isolde* subsp. *isolde* (Banks, 1945) †: LL (Trujillo)

Subgenus: *Ceropales* Latreille, 1796

*basirufus* Rohwer, 1913: Endemic, CU (Santa Ana)

***Irenangelus* Schulz, 1906**

*furtivus* Evans, 1969: MD (Avispas)

*lucidus* Evans, 1969: HU (Tingo María), MD (Avispas, Pakitza)

*ichneumonoides* Ducke, 1908: LO (Iquitos), MD (Avispas, Pakitza), UC (Pucallpa)

*reversus* (Smith, 1873): HU (Pachitea), MD (Avispas), UC† (B. Abad).

**Epipompilinae**

***Epipompilus* Kohl, 1884**

*inca* Evans, 1967: Endemic, CU (Machu Picchu)

*innubus* Evans, 1961: Endemic, HU (Cuchara)

*williamsi* (Banks, 1947)†: PA (Oxapampa).

**Pepsinae**

**Ageniellini**

***Ageniella* Banks, 1912**

Subgenus: *Alasagenia* Banks, 1944

*corymele* Banks, 1946: JU (Chanchamayo)

*fortipes* (Smith, 1873) †: UC (Pucallpa)

*pilifrons* (Cameron, 1912) †: HU (Monzon- Rondos River), JU (Chanchamayo), UC (B. Abad, Pucallpa).

Subgenus: *Ageniella* Banks, 1912

*ruficeps* (Smith, 1864) †: UC (Pucallpa)

Subgenus: *Ameragenia* Banks, 1945

*alcimeda* Banks, 1946: Endemic, JU (El Campamento)

*pretiosa* Banks, 1946: JU (Chanchamayo)

Subgenus: *Cyrtagenia* Evans, 1973

*fallax* (Arlé, 1947): MD (Avispas)

Subgenus: *Lissagenia* Banks, 1946

*difformis* Banks, 1944: LO (Iquitos)

Subgenus: *Priophanes* Banks, 1944

*plagosa* Banks, 1946: PA (Puerto Bermúdez)

***Auplopus* Spinola, 1841**

*caeruleosoma* Banks, 1946: Endemic, LI (Lima)

*deceptor* (Smith, 1873) †: HU (Cord. Azul)

*eriodes* Dreisbach, 1963: Endemic, PI (Piura)

*lasios* Dreisbach, 1963: Endemic, HU (Tingo María)

*peruana* Banks, 1946: Endemic, LI (Matucana, Santa Eulalia)

*striatus* Dreisbach, 1963: Endemic, LI (Lima, Verrugas)

***Mystacagenia* Evans, 1973** [Evans 1973b]

*albiceps* Evans, 1973: MD (Avispas, Pakitza)

*bellula* Evans, 1973: MD (Avispas)

***Phanochilus* Banks, 1944** [Banks 1946]

*gloriosus* (F. Smith, 1873)

*nobilitatus* F. Smith, 1864: LO (Itaya), PA (Puerto Bermúdez)

*ornatus* Banks, 1946: Endemic, SM (Achinamisa)

***Priocnemella* Banks, 1925** [Banks 1946]

*delila* (Banks, 1944): MD (Pakitza)

*fairchildi* (Banks, 1925): MD (Pakitza)

*hexagona* subsp. *omissa* Banks, 1946: HU† (Tingo Maria), JU† (Chanchamayo), UC† (Atalaya, Pucallpa)

*rufothorax* (Banks, 1925): MD (Pakitza)

**Pepsini**

***Adirostes* Banks, 1946**

*ariphana* Roig-Alsina, 1984: HU (Carpish)

*tolteca* Banks, 1946: Endemic, JU (Casa de patos, Palca)

*wahisi* Roig-Alsina, 1984: LL (Choquisongo)

*willinki* Roig-Alsina, 1984: AN (Conococha)

***Aimatocare* Roig-Alsina, 1989** [Evans 1968b, Roig-Alsina 1989]

*longula* (Banks, 1946): CU (Paucartambo, Quincemil), MD (Avispas)

*vitrea* (Fox, 1897): SM (Achinamisa)

***Caliadurgus* Pate, 1946** [Dreisbach 1961b]

*pretiosus* (Fox, 1897) †: PA (Oxapampa).

*ornatus* (Dreisbach, 1961): Endemic, HU (Monzón)

***Entypus* Dahlbom, 1843** [Banks 1946, Roig-Alsina 1981]

*bituberculatus* (Guérin-Méneville, 1838): SM (Achinamisa)

*caerulans* (Lepeletier, 1845) (= *mammillatus* (Fox, 1897)): JU (Chanchamayo, El Campamento).

*decoloratus* (Lepeletier, 1845) †: HU (Monzón).

*dumosus* (Spinola, 1851) †: LA (Jayanca), LI (Matucana), LL (Simbal).

*fossulatus* (Giner Marí, 1944): LI (Matucana)*.*

*gigas* (Fabricius, 1804) †: UC (Pucallpa).

*molestus* (Banks, 1946): Endemic, CA (Q Limón), HU (Huánuco), LA† (Lambayeque), LI (Lima, Matucana†), LL† (Simbal)

*nitidus* (Banks, 1946): HU† (Cayumba), JU (Chanchamayo)

*peruvianus* (Rohwer, 1913): CU (Machu Picchu†, Apurimac River†, Santa Ana, Urubamba River†), JU (Chanchamayo)

***Minagenia* Banks, 1934** [Banks 1946]

*peruana* (Banks, 1946): Endemic, JU (Chanchamayo)

***Pepsis* Fabricius, 1804**

*auriguttata* Burmeister, 1872: CA, JU, LO, MD, UC

*purpureipes* Packard, 1869: JU

*sabina* Mocsáry, 1885: CA, JU, LO, SM

*chiliensis* Lepeletier, 1845: AR, LA, LI (Lima, Matucana),

*deaurata* Mocsáry, 1894: HU, JU, LO (Iquitos)

*elevata* Fabricius, 1804: CU, UC

*lycaon* Banks, 1945: CA (Jaén), LA, LI

*terminata* Dahlbom, 1843: AP, AY (Ninabamba), CA (Jaén), CU, HU (Iparía), JU, LO, SM, UC

*elongata* Lepeletier, 1845: LO

*grossa* (Fabricius, 1798): CA, JU (Chanchamayo), LA, LO, PI, SM, TU

*marthae* Vardy, 2002: Endemic, CU (Machu Picchu), JU (Chanchamayo)

*pulawskii* Vardy, 2002: Endemic, AN (Huaylas), CA (Sunchubamba), LA (Chinche, Olmos)

*tolteca* Lucas, 1895: AM, AP, AY (Ayacucho), CA, CU, HU (Iparía, Tingo María), JU (La Merced), LI (Matucana, San Bartolomé, San Mateo), LL

*asteria* Mocsáry, 1894: HU, LO (Iquitos, Yurimaguas), MD, SM, UC

*crassicornis* Mocsáry, 1885: LO

*fumipennis* Smith, 1855: LO (Iquitos), SM (Cumbasa)

*inclyta* Lepeletier, 1845: CA (Celendin), LO, PI, UC,

*luteicornis* Fabricius, 1804: LO (Itaya), UC, (=alector Banks, 1946)

*sommeri* Dahlbom, 1845

*vitripennis* Smith, 1855: CA, CU, HU (Huallaga), JU (Chanchamayo), LI, LO (Iquitos), MD, PI, SM, UC

*xanthocera* Dahlbom, 1843: CA, CU, LO (Iquitos)

*cyanescens* Lepeletier, 1845: CA, CU, LO

*lampas* Lucas, 1895: AP, AY, MD

*multichroma* Vardy, 2005: AM (Bagua), CA (Jaén), LA (Chiclayo), PI (Piura)

*menechma* Lepeletier, 1845: CA, HU, JU (Huacapistana), LO, MD, PA, SM (Cumbasa, Tarapoto), UC

*montezuma* Smith, 1855: AM (Bagua), AP, AR, AY, CA, CU (Machu Picchu), HU, IC, JU (Huacapistana), LA, LI (Lima, Matucana), LL (Otuzco), LO, PA, PI, SM, UC, (“Yucay”)

*egregia* Mocsáry, 1885: JU, LO (Iquitos), MD (Pakitza)

*cooperi* Vardy, 2000: AM (Bagua), CA (Jaén, Pomahuaca), LA (Olmos)

heros (Fabricius, 1798): JU, LO, MD, SM, UC

*petitii* Guérin-Méneville, 1831: LA, LI (Matucana), LL, PI, TU

*vinipennis* Packard, 1869: AM, HU, LO (Iquitos), SM (Moyobamba),

*festiva* Fabricius, 1804: HU, JU, LO, MD (Pakitza), PA, SM, UC

*gracilis* Lepeletier, 1845: HU, LO (Iquitos), SM

*hyalinipennis* Mocsáry, 1885: HU, JU, LO (Pebas, Yurimaguas), SM

*infuscata* Spinola, 1841: JU (La Merced), LO (Iquitos), SM (Achinamisa, Moyobamba)

*krombeini* Vardy, 2005: Endemic, LO (Pebas)

*purpurea* Smith, 1873: JU, LO (El Encanto, Putumayo), SM (Achinamisa),

*willinki* Vardy, 2005: AM (Santiago), CU (Atalaya), HU (Monzón, Sinchona, Tingo María), PA (Puerto Bermúdez), UC (Atalaya)

*seladonica* Dahlbom, 1843: MD, UC

*sumptuosa* F. Smith, 1855

*apicata* Taschenberg, 1869: LO

*aurozonata* Smith, 1855: LO, UC

*plutus* Erichson, 1849: MD (La Torre), UC

*pulszkyi* Mocsáry, 1885: AM, HU, JU, LO, SM

*toppini* Turner, 1915: CA, HU (Huallaga), JU (Chanchamayo, Perené, Satipo), MD, PA, UC

*dayi* Vardy, 2005: CU (Atalaya), UC (B. Abad)

*hirtiventris* Banks, 1946: HU, LO (Iquitos, Mishuyacu, San Roque), SM (Achinamisa)

*ianthina* Erichson, 1848: CA, LI, LO (Iquitos, Pebas)

*nana* Mocsáry, 1885: AM, AP, AY (Ninabamba), CA, CU, HU, JU (Chanchamayo, Perené), LO (Iquitos, La Chorrera, La Sombra), MD, PA (Puerto Bermúdez), SM, UC (Yarinacocha)

*viridis* Lepeletier, 1845: MD.

***Pompilocalus* Roig-Alsina, 1989**

*carrascoi* Roig-Alsina, 1989: Endemic, CU (Acomayo, Chocco, Urubamba)

*edmondii* (Brèthes, 1924): AP (Andahuaylas), AR (Arequipa), AY (Lucanas, Quinua), CU ( Acomayo, Chocco, Cusco, Lucre, Machu Picchu), LI (Matucana), LO (Iquitos), PU (Arapa, Chucuito, Chuquibambilla, Puno, Sillustani, Tirapata)

*hirticeps* (Guérin-Méneville, 1838): TA (Tacna)

*huaynacapac* Roig-Alsina, 1989: CU (Chocco)

*mancocapac* Roig-Alsina, 1989: Endemic, LI (Huamantanga)

*maytocapac* Roig-Alsina, 1989: Endemic, CU (Acomayo, Chocco, Cusco, Pisaq, Urubamba)

*pachacutec* Roig-Alsina, 1989: Endemic, AP (Andahuaylas), AR (Arequipa), AY (Tambo), CU (Acomayo, Capana, Chocco, Cusco, Limatambo, Lucre, Paucartambo, Sacsayhuaman, San Sebastián, Urubamba), JU (Acolla, Tarma-Jauja), LI Huamantanga, Tambo)

*tacaynamo* Roig-Alsina, 1989: Endemic, LL (Choquisongo)

*tupacyupanqui* Roig-Alsina, 1989: Endemic, JU (Acolla, Satipo), CU (Acomayo, Chocco, Cusco)

***Priocnemis* Schiödte, 1837** [Dalla Torre 1897]

*dichrous* Dalla Torre, 1897: (=bicolor Taschenberg, 1880)

***Priocnessus* Banks, 1925** [Dreisbach 1960, 1961a]

*grandis* Dreisbach, 1961: Endemic, UC (Ucayali)

*prominens* Banks, 1945

*semirufus* Dreisbach, 1960: Endemic, CA (Huascaray)

**Pompilinae**

**Aporini**

***Aporus* Spinola, 1808**

Subgenus: *Aporus* Spinola, 1808

*cuzco* Evans, 1973: CU (Cusco, Chocco, Lucre, Urubamba Rvier†)

Subgenus: *Neoplaniceps* Bradley, 1944

*umbratilis* Evans, 1966: CU (Lucre, Quincemil, Chocco), HU† (Tingo Maria)

Subgenus: *Notoplaniceps* Bradley, 1944

*canescens* Smith, 1873: HU (Tingo María), MD (Avispas, Upper Madre de Dios River†)

***Euplaniceps* Haupt, 1930** [Bradley 1944]

*ceres* (Cameron, 1897): MD (Pakitza)

*varia* Bradley, 1944: CU† (Atalaya), LO (San Roque), UC† (B. Abad)

**Pompilini**

***Agenioideus* Ashmead, 1902**

Subgenus: *Enbanksia* Evans, 1965

*minutus* (Banks, 1947): MD (Avispas)

***Anoplius* Dufour, 1834**

Subgenus: *Anopliodes* Banks, 1939

*varius* (Fabricius, 1804): HU† (Cord. Azul, Tingo Maria), UC† (B. Abad)

Subgenus: *Anoplius* Dufour, 1834

*ambatoensis* (Cameron, 1903): CU† (Urubamba), LI† (Chosica)

*fulgidus* (Cresson, 1865): PA (Puerto Bermúdez)

*minor* Banks, 1947: Endemic, AR (Arequipa)

Subgenus: *Arachnophroctonus* Howard, 1901

*arequipensis* (Brèthes, 1924): AR (Arequipa)

*atrimene* Banks, 1947: LI

*escomeli* (Brèthes, 1912): Endemic, AR (Sabandia)

*inculcatrix* (Cameron, 1912): JU (El Campamento, Chanchamayo†), LO† (Est. Jenaro Herrera)

*marginicollis* (Taschenberg, 1869): PA (Puerto Bermúdez)

*peruviana* Banks, 1947: Endemic, PA (Victoria), PU (Puno)

Subgenus: *Notiochares* Banks, 1917

*amethystinus* subsp. *amethystinus* (Fabricius, 1793)

*amethystinus* subsp. *exclusus* (Smith, 1873)

*diffinis* Banks, 1947: Endemic, LO (Iquitos)

***Arachnospila* Kincaid, 1900**

*dichromorphus* (Rohwer, 1913): Endemic, CU (Cusco), LI (Pativilca), JU (La Oroya)

*titicacaensis* (Strand, 1911): Endemic, PU (Puno)

***Aridestus* Banks, 1947**

*porteri* Evans, 1966: CU (Cusco)

***Austrochares* Banks, 1947**

*elsinore* Banks, 1947: Endemic, CL† (Ventanilla), LA† (Chiclayo, Jayanca, Lambayeque, Olmos), LI (Lima), LL† (Simbal, Trujillo)

***Balboana* Banks, 1944**

*auripennis* (Fabricius, 1804): CU† (Quincemil), HU† (Tingo Maria), MD (Pakitza), UC† (Atalaya, Pucallpa)

*fraternus* Banks, 1946: Endemic, LI (Lima)

*manifestata* (Smith, 1864) †: LO (Est. Jenaro Herrera)

***Episyron* Schiödte, 1837**

*conterminus* subsp. *conterminous* Smith, 1873: LI (Lima)

***Evagetes* Lepeletier, 1845**

*peruana* Banks, 1947: Endemic, LL (Trujillo), PA (Victoria)

*copiosus* Banks, 1947†: CU (Cusco)

***Paracyphononyx* Gribodo, 1884**

*incalis* Banks, 1947: Endemic, AY (Ninabamba), LO (La Sombra)

*unicolor* (Smith, 1879) †: CU (Urubamba River, Apurimac River, Machu Picchu), HU (Tingo María), JU (Chanchamayo)

***Poecilopompilus* Howard, 1901**

*apicalis* (Banks, 1947): JU (Perené)

*decedens* (Smith, 1873): LO (Iquitos)

*rubricatus* (Smith, 1879): LA† (Chiclayo, Jayanca, Lambayeque), LI† (Cieneguilla, Lima), LL† (Trujillo, Jequetepeque River), PI (Negritos)

***Priochilus* Banks, 1944**

*captivum* (Fabricius, 1804) †: LO (Contamana, Iquitos), PA (Pichis), UC (Pucallpa)

*formosus* Banks, 1944: CU† (Quincemil), JU (Chanchamayo†, Perené), SM† (Nuevo Progreso).

*gloriosum* subsp. *multifasciatum* (Taschenberg, 1869)

*gloriosum* subsp. *gloriosum* (Cresson, 1869) †: JU (Chanchamayo), UC (B. Abad, Pucallpa)

*gracillimus* (Smith, 1855)(=*scrupulum* (Fox, 1897)): HU† (Tingo Maria), MD (Pakitza), UC† (Pucallpa)

*nobilis* (Fabricius, 1787): JU (El Campamento)

*pectoralis* (Smith, 1855) (=*imperius* Banks, 1944): CA† (Jaén), LO (Est. Jenaro Herrera†, Iquitos, Itaya, La Sombra), MD† (Atalaya), UC† (Pucallpa).

*peruanus* Banks, 1946: Endemic, LO (Iquitos, Mishuyacu)

*regius* subsp. *infumatus* Banks, 1946: AM (Santiago), HU (Upper Río Huallaga), LO (Iquitos), SM (Achinamisa)

*regius* subsp. *regius* (Fabricius, 1804): AM (Santiago), HU† (Cayumba, Tingo Maria, Yanayacu), JU† (Chanchamayo), UC (Atalaya†, B. Abad†, San Alejandro, Pucallpa†)

*ruficoxalis* (Fox, 1897) †: UC (Pucallpa)

*sericeifrons* (Fox, 1897): CU† (Quincemil), HU† (Monzon- Rondos River, Tingo Maria), MD (Pakitza), UC† (B. Abad, Pucallpa)

*splendidulum* subsp. *splendidulum* (Fabricius, 1804): HU† (Tingo Maria, Cord. Azul), LO† (Est. Jenaro Herrera), MD (Pakitza), UC† (B. Abad, Pucallpa)

*superbus* Banks, 1944: LO (Iquitos), SM (Achinamisa)

*veraepascis* (Cameron, 1893): LO† (Est. Jenaro Herrera), MD (Pakitza)

***Tachypompilus* Ashmead, 1902** [Banks 1947]

*pallidus* Banks, 1947: Endemic, LI (Chosica†, Matucana), LL† (Simbal)

*xanthopterus* (Rohwer, 1913): CU (San Miguel)

**Notocyphinae**

***Notocyphus* Smith, 1855** [Banks 1947]

*brevicornis* Fox, 1897: Endemic?, HU (Huánuco)

*crassicornis* Smith, 1864†: JU (Chanchamayo), LO (Estación Jenaro Herrera), UC (Pucallpa).

*laetabilis* (Smith, 1873) †: UC (Pucallpa).

*maculifrons* Smith, 1873: Endemic?, LO (Iquitos).

*multipicta* Smith, 1873†: LO (Est. Jenaro Herrera), UC (B. Abad, Pucallpa)

*pallidipennis* Banks, 1947: Endemic, AM (Santiago)

*saevissimus* Smith, 1855†: UC (Pucallpa)

*signatus* Banks, 1947: Endemic, LO (La Sombra)

*thetis* Banks, 1945: CU† (Atalaya), UC† (Pucallpa)

*tyrannicus* Smith, 1855: LO (Iquitos)

*vindex* Smith, 1864: LO (Iquitos)

**SCOLIIDAE**

**Campsomerinae**

***Campsomeris* Guérin-Méneville, 1839**

Subgenus: Aelocampsomeris Bradley, 1957

*brethesi* (Bradley, 1927): Endemic, LO (San Roque)

*variegata* Fabricius, 1793: JU† (Chanchamayo)

Subgenus: *Campsomeris* Guérin-Méneville, 1839

*peregrina* (Lepeletier, 1845)

*regifica* Bradley, 1945: HU (Huallaga)

*servillei* Guérin-Méneville, 1838: LI

Subgenus: *Dielis* de Saussure & Sichel, 1864

*chilensis* (de Saussure & Sichel, 1864): CU (Santa Ana), LI (Lima).

*dorsata* Fabricius, 1787: LO (Iquitos)

*whitelyi* Kirby, 1889: CU (Tambo)

Subgenus: *Lissocampsomeris* Bradley, 1957

*arneohirta* (Fox, 1896) †: JU (Chanchamayo), UC (B. Abad)

*wesmaeli* (Lepeletier, 1845) †: CU (Atalaya), HU (Monzon – Rondos River), JU (Chanchamayo), UC (B. Abad, Pucallpa)

**Scoliinae**

***Scolia* Fabricius, 1775**

Subgenus: *Hesperoscolia* Bradley, 1974

*rufiventris* (Fabricius, 1804) †: JU (Chanchamayo), UC (B. Abad, Pucallpa)

**VESPIDAE**

**Eumeninae**

**Eumenini**

***Alphamenes* van der Vecht, 1977**

*campanulatus* (Fabricius, 1804): HU (Monzón, Tingo María), JU (Chanchamayo, El Campamento), UC (Pucallpa)

*semiplanus* Giordani Soika, 1978: HU (Llullapichis), LO (Iquitos), UC (Pucallpa)

***Cyphomenes* Giordani Soika, 1978**

*infernalis* subsp. *infernalis* (de Saussure, 1875): HU (Llullapichis), UC (Pucallpa)

*infernalis* subsp. *weyrauchi* Giordani Soika, 1978: JU (Satipo)

***Eumenes* Latreille, 1802**

Subgenus: *Zeteumenoides* Giordani Soika, 1972

*filiformis* (de Saussure, 1855): LO (Iquitos), MD†, UC (Pucallpa)

*versicolor* de Saussure, 1852: HU (Llullapichis)

***Minixi* Giordani Soika, 1978**

*brasilianum* (de Saussure, 1978): HU (Llullapichis, Tingo María)

***Omicron* de Saussure, 1855**

*aequale* Giordani Soika, 1978: HU (Previsto, Tingo María)

*belti* (Cameron, 1907): CU (Callanga)

*conclamatum* Giordani Soika, 1978: HU (Tingo María), UC (Pucallpa)

*criticum* subsp. *criticum* (Schulz, 1906): CU (Quincemil), HU (Tingo María), JU (Chanchamayo, El Campamento), PA (Puerto Bermúdez), UC (Atalaya, Pucallpa)

*criticum* subsp. *richardsi* Giordani Soika, 1978: CU (Vilcanota), SM

*foxi* (Schulz, 1905): LO (La Sombra)

*globicolle* (Spinola, 1841): CU (Callanga), HU (Llullapichis, Pachitea), JU (Chanchamayo), LO (La Chorrera, Putumayo), UC (Pucallpa)

*gondwanianum* Giordani Soika, 1978: CU (Machu Picchu), HU (Divisoria, Sinchona), JU (El Campamento, Huacapistana, Oreja de Capelo)

*lustratum* Giordani Soika, 1978: UC (Pucallpa)

*nanum* subsp. *incarum* Giordani Soika, 1978: CU (Callanga, Quincemil), HU (Previsto, Tingo María), JU (Chanchamayo, El Campamento), LO (Iquitos), MD (Avispas), UC (Atalaya, Pucallpa)

*nymphale* (Zavattari, 1912): HU (Tingo María), JU (Chanchamayo)

*paranymphum* (Zavattari, 1912): HU ( Previsto, Tingo María), JU (El Campamento), UC (Pucallpa, Previsto†)

*reliquum* Giordani Soika, 1978: JU (Oreja de Capelo)

*rubellulum* subsp. *flavellulum* Giordani Soika, 1978: CA (Tembladera), LL (Chepén), LI (Chosica)

*ruficolle* subsp. *schunkei* Giordani Soika, 1978: JU (Chanchamayo)

*ypsilon* Giordani Soika, 1978: CU (Vilcanota)

***Pachymenes* de Saussure, 1852**

*ghilianii* Spinola, 1851: HU (Tingo María), LO (Iquitos, Pebas), MD† (=*ghilianii* subsp. *olivaceus* (de Saussure, 1875), =*peruanus* Schrottky, 1911, see in Grandinete *et al.* 2014)

*consuetus* Giordani Soika, 1990: JU† (Chanchamayo), MD (Avispas, Pucallpa†), UC (B. Abad) (=*obscurus* subsp. *consuetus* Giordani Soika, 1990, see in Grandinete *et al.*, 2014)

*novarae* (de Saussure, 1867): CU (Callanga, Vilcanota), HU (Llullapichis, Monzón, Tingo María), UC (Pucallpa) (=*Santamenes novarae* (de Saussure, 1867), see in Grandinete *et al.* 2014)

*orellanae* (Schulz, 1905): HU (Llullapichis, Tingo María†), JU (Chanchamayo), LO† (Iquitos), MD† (Res. Nac. Tambopata), UC (B. Abad†, Pucallpa) (=*orellanae* subsp. *orellanae* (Schulz, 1905), = *orellanae* subsp. *vardyi* Giordani Soika, 1990, see in Grandinete *et al.*, 2014)

*peregrinus* (Zavattari, 1912): CU (Callanga), HU (Llullapichis), LO (Pebas) (=*Santamenes peregrinus* (Zavattari, 1912), see in Grandinete *et al.* 2014)

***Pachyminixi* Giordani Soika, 1978**

*bifasciatum* (von Schulthess, 1904): CU (Cusco, Lucre)

***Pararhaphidoglossa* von Schulthess, 1910**

*bicarinata* Giordani Soika, 1978: HU (Pachitea), UC (Pucallpa)

*carpenteri* Cooper, 2013: Endemic, LO (Iquitos)

*colorata* (Fox, 1899): JU (Chanchamayo, El Campamento), UC (Pucallpa)

*gribodoi* (Zavattari, 1912): JU (Pan de Azucar)

*mestiza* Giordani Soika, 1978: HU (Yanayacu)

*imitatrix* Giordani Soika, 1978: LO (La Chorrera), UC (Pucallpa)

*invenusta* Giordani Soika, 1978: HU (Tingo María), LO (Mishuyacu, San Roque)

*schunkei* Cooper, 2014: UC (B. Abad)

*sulcata* Cooper, 2013: LO (Iquitos)

***Pirhosigma* Giordani Soika, 1978**

*mearimense* subsp. *putumayense* Giordani Soika, 1978: LO (Putumayo)

*mearimense* subsp. *mearimense* (Zavattati, 1912) †: UC (Pucallpa)

***Sphaeromenes* Giordani Soika, 1978**

*discrepatus* Giordani Soika, 1978 Endemic: LI (Chillón, Rímac, Santa Rosa de Quives†), LL† (Trujillo).

***Stenosigma* Giordani Soika, 1978**

*humerale* Giordani Soika, 1990: JU (Chanchamayo), PA (Dos de Mayo, Pichis)

*testaceum* (Fox, 1899): UC (Pucallpa)

***Zeta* de Saussure, 1855**

*argillaceum* (Linnaeus, 1758): AP (Abancay-Cusco), CA (Jaén), HU (Huánuco), JU (Chanchamayo, El Campamento, Huacapistana), LI (Chosica, Lima, Verrugas), PI, UC (Pucallpa)

**Odynerini**

***Ancistrocerus* Wesmael, 1836**

*epicus* (Zavattari, 1912) Endemic: LI (San Paulo)

*pilosus* de Saussure, 1855: AN (Huaráz), PA (Huancabamba)

***Brachymenes* Giordani Soika, 1961**

*dyscherus* subsp. *dyscherus* (de Saussure, 1852): CU (Callanga), HU (Llullapichis), JU (Chanchamayo), UC (B. Abad, Cord. Azul†, Pucallpa)

*wagnerianus* (de Saussure, 1875): JU (Chanchamayo), UC† (Cord. Azul)

***Cephalastor* Giordani Soika, 1982**

*chasqui* Garcete Barrett, 2001: JU (Chanchamayo)

*lambayeque* Garcete Barrett, 2002 Endemic: LA (Lambayeque)

*tupasy* Garcete Barrett, 2001Endemic: MD (Puerto Maldonado, Tambopata)

***Gamma* Zavattari, 1912**

*ventricosum* subsp. *peruvianum* (Zavattari, 1912) Endemic: CU (Vilcanota)

*ventricosum* subsp. *ventricosum* (de Sauss ure, 1852): PA (Huancabamba)

***Hypalastoroides* de Saussure, 1852**

Subgenus: *Hypalastoroides* de Saussure, 1852

*argentinus* (Brèthes, 1903)

*brasiliensis* (de Saussure, 1856): JU (Satipo), LO† (Iquitos)

***Hypodynerus* de Saussure, 1855**

*akros* Willink, 1970 Endemic: JU (Casapalo).

*andeus* (Packard, 1869): AN (Huaráz, Recuay), AR (Arequipa, Cotahuasi, Tiabaya, Yura), CU (Cusco, Miravalle, Ollantaytambo, San Sebastián, Vilcanota), LI (Canta, Miñita, Matucana, San Mateo, Santa Eulalia, Verrugas), LL (Otuzco), TA (Tacna).

*arequipensis* (du Buysson, 1913): AR (Arequipa), JU†.

*chiliotus* de Saussure, 1852: AR (Arequipa), CU (Callanga)

*dimidiaticornis* Zavattari, 1912 Endemic: CU (Marcapata)

*foersteri* Giordani Soika, 1961: PU (Camacani, Chimú, Crucero Alto, La Huerta, Puno, Urcunimuni)

*huancabambae* Schrottky, 1911 Endemic: PA (Huancabamba)

*joergenseni* Schrottky, 1909

*nigricornis* Rohwer, 1913: CU (Cusco), JU (La Oroya)

*obscuripennis* (Spinola, 1851): AR† (Arequipa)

*rufinodis* (du Buysson, 1913) Endemic: AR (Arequipa), TA (Tarata)

*tarabucensis* (de Saussure, 1855): PU (Puno)

*vestitus* (de Saussure, 1855): CU

***Incodynerus* Willink, 1968**

*alticola* Willink, 1969: AR, CU (Cusco), PU (Amantaní, Camacani, La Huerta, Puno)

*ambiguus* Willink, 1969 Endemic: AP (Andahuaylas)

*coccineipes* (Zavattari, 1912): CU (Cusco, Lucre, Vilcanota)

*melanotrichus* Willink, 1969: LI (Pativilca)

*romandinus* (de Saussure, 1852): CA (Jaén), CU (Tincochaca), PA (Huancabamba)

*tegularis* (Schrottky, 1911) Endemic: AN (Huaráz), AP (Abancay), CA (Cutervo), CU (Cusco, Lucre), LI (Matucana), PA (Huancabamba),

*urubambae* (Schrottky, 1911): CU (Urubamba)

*vilcanotae* (Zavattari, 1912): CU (Cusco, Vilcanota), LA (Olmos (2000m)), PU (Camacani, La Huerta)

***Monobia* de Saussure, 1852**

*angulosa* de Saussure, 1852: AP (Abancay-Cusco), AY (Ninabamba), CU (Ocobamba, Vilcanota), JU (Chanchamayo, Pampa Hermosa), LO (Iquitos), SM, UC (Pucallpa)

*atrorubra* Ducke, 1904: LO (Iquitos)

*caliginosa* Willink, 1982: PA (Huancabamba)

*cyanipennis* (Guérin-Méneville, 1831): AN (Huallanca), AR (Camaná), LA (Chongoyape, Lambayeque, Oyotun, Zaña), LI (Atocongo, Lima, Q. Verde, Santa Rosa de Quives), LL (San Pedro de Lloc), PI (Lobitos, Negritos, Paita), TU (Tumbes)

*deplanata* Ducke, 1908: LO (Mishuyacu)

*funebris* Gribodo, 1891: CU (Paucartambo), HU (Llullapichis, Tingo María), JU (Chanchamayo), LO (Yurimaguas)

*incarum* Bequaert, 1912: AR (Tambo), CA (Sunchubamba), LL (Chepén, Lambayeque), LI (Barranca, Chorrillo s, Lima, Santiago de Surco, Verrugas), LL (Simbal)

***Montezumia* de Saussure, 1852**

*analis* de Saussure, 1855: HU (Llullapichis), JU (Satipo), LO (Iquitos, Mishuyacu), SM (Achinamisa), UC (San Alejandro)

*azurescens* (Spinola, 1851): AP, CU (Callanga, Vilcanota), HU (Llullapichis, Monzón, Pachitea), JU (Pan de Azúcar, Satipo), LI (Callao), LO (Iquitos, Mishuyacu), UC (Atalaya, Pucallpa)

*coeruleorufa* Willink, 1982: CU (Callanga), HU (Tingo María), LO (Mishuyacu)

*dimidiata* de Saussure, 1852: HU (Llullapichis, Sinchona, Tingo María), JU (Chanchamayo, Pan de Azúcar, Satipo), LO (Iquitos, Mishuyacu), SM (Bellavista), UC (Pucallpa)

*grossa* Willink, 1982: UC (Neshuya)

*liliacea* Gribodo, 1891: HU (Tingo María), JU, UC (Pucallpa)

*liliaciosa* Gribodo, 1891: LO (Pebas)

*morosa* de Saussure, 1852: JU (Satipo)

*pelagica* subsp. *pelagica* de Saussure, 1852: AM (Marañón), HU (Tingo María), LO (Iquitos), UC (La Junta, Pucallpa)

*pelagica* subsp. *sepulchralis* de Saussure, 1855: HU (Llullapichis), UC (Pucallpa)

*petiolata* de Saussure, 1855: HU (Tingo María)

*trinitata* Willink, 1982: SM

*vechti* Willink, 1982: CU (Callanga), HU (Cueva de las Pavas, Pachitea), LO (Iquitos), MD (Iberia)

***Pachodynerus* de Saussure, 1870**

*argentipilis* Willink & Roig-Alsina, 1998: HU (Monzón)

*bicuspidatus* Willink & Roig-Alsina, 1998: LI (Chosica, Lima, Rímac), LA (Lambayeque, Zaña), LI (Chancay), LL (Chepén, Chicama, Samne, Simbal, Trujillo)

*brevithorax* (de Saussure, 1853): HU (Tingo María), LA (Olmos), LO (Iquitos, Tapiche), UC (Pucallpa, Yarinacocha)

*diabolicus* (de Saussure, 1853) Endemic: AN (Casma), IC (Pisco), LA (Lambayeque, Zaña), LI (Callao, Cocachacra, Cupiche, La Molina, Matucana, Palle, Santa Eulalia, Surco, Verrugas), LL (Chepén, Laredo, Simbal, Trujillo), PI (Negritos, Paita, Talara)

*gaullei* Brèthes, 1920: CA (Q Nancho), LA (Chongoyape, Lambayeque, Motupe, Oyotum, Olmos, Zaña), LL (Chepén, Pacasmayo, Samne, Simbal, Trujillo), LI (Chancay, Chosica, Cupiche, Palle, Santa Eulalia), PI (Mogollón, Rumipampa), TU (Puerto Pizarro)

*gianellii* (Gribodo, 1891): HU (Llullapichis, Tingo María)

*grandis* Willink & Roig-Alsina, 1998: LL (Simbal)

*guadulpensis* (de Saussure, 1853): AP (Curahuasi), AY (Cangallo, Huanta, Ninabamba), CA (Jaén), CU (Quillabamba, Santa Ana), HU (Las Palmas, Llullapichis, Monzón, Tingo María, Yurac), JU (Chanchamayo, Huancayo, La Merced, Perené, San Luis de Shuaro), MD (Tambopata), PA (Huancabamba), UC (Acobamba, Pucallpa)

*laplatae* (de Saussure, 1870): AY (Huatatas)

*lima* Willink & Roig-Alsina, 1998: CA (Carahuasi, Q Nancho), LA (Lambayeque), LI (Chosica, Cupiche, Lima, Palle, Surco, Santa Eulalia, Verrugas), LL (Cartavio, Chicama, Samne, Simbal, Trujillo), TU (Tumbes)

*nasidens* (Latreille, 1817): CU (Cosñipata), HU (Cueva de las Pavas, Llullapichis, Previsto, Tingo María), JU (Chanchamayo), LA (Lambayeque), LI (Chosica), LL (Cartavio), MD (Tambopata), UC (Pucallpa, San Alejandro)

*pannus* Willink & Roig-Alsina, 1998: LA (Lambayeque)

*parachartergoides* (Ducke, 1911): HU (Cueva de las Pavas)

*peruensis* (de Saussure, 1855): AR (Arequipa, Sabandia, Tiabaya, Tingo, Yura), CA (Querocotillo), CU (Callanga, Cusco, Huaisampillo), IC (Huacachina), LA (Chongoyape, Motupe, Olmos), LI (Barranca, Chosica, Cupiche, La Molina, Lima, Matucana, Pachacámac, Palle, Surco, Santa Eulalia, Verrugas), LL (Cartavio, Chicama, Simbal, Trujillo), PI (Negritos, Paita, Piura, Talara), PU (La Huerta, Santo Domingo), TA (Tacna)

*ucayali* Willink & Roig-Alsina, 1998: UC (San Alejandro)

***Parazumia* de Saussure, 1855**

*carinulata* (Spinola, 1851): CU (Callanga), LO (Mishuyacu), UC (Pucallpa)

***Pseudodynerus* de Saussure, 1855**

*maxillaris* (de Saussure, 1875): CU, JU (Chanchamayo), UC (Pucallpa)

*subapicalis* (Fox, 1902): HU (Tingo María, Huánuco†), JU (Perené)

***Stenodynerus* de Saussure, 1863**

*corallineipes* (Zavattari, 1912) Endemic: AN (Caraz)

*inca* de Saussure, 1871: LI

*mimeticus* Bertoni, 1918: AR (Arequipa), CU (Barrio Magisterial, Lucre, Pisaq)

***Stenonartonia* Giordani Soika, 1973**

*apicipennis* (Fox, 1902): CU (Callanga)

*polybioides* (von Schulthess, 1904): CU (Callanga), HU (Llullapichis), LO (Iquitos)

***Symmorphus* Wesmael, 1836**

Subgenus: *Symmorphus* Wesmael, 1836

*cristatus* de Saussure, 1855

**Zethini**

***Zethus* Fabricius, 1804**

Subgenus: *Zethoides* Fox, 1899

*biglumis* subsp. *biglumis* Spinola, 1841: LO (Iquitos)

*binodis* (Fabricius, 1793): LO (Putumayo)

*carinatus* F. Smith, 1857: JU (El Campamento)

*peruvicus* R. Bohart & Stange, 1965 Endemic: LO (Iquitos)

*pygmaeus* Zavattari, 1912: LO (Pebas), HU (Monzón)

*schadei* R. Bohart & Stange, 1965: JU (Perené)

*schlingeri* R. Bohart & Stange, 1965 Endemic: UC (Pucallpa)

*toltecus* subsp. *toltecus* de Saussure, 1875: HU (Tingo María), LO (Pebas)

Subgenus: *Zethus* Fabricius, 1804

*adonis* R. Bohart & Stange, 1965: HU (Tingo María)

*alticola* R. Bohart & Stange, 1965 Endemic: CA (Cutervo, Cajamarca), LI (Callahuanca, Churín, Matucana, Palle), LL (Samne)

*attenuatus* R. Bohart & Stange, 1965 Endemic: HU (Monzón)

*boharti* Stange, 1976 Endemic: LO (Iquitos)

*caracis* R. Bohart & Stange, 1965 Endemic: SM (Achinamisa)

*cataractae* Cooper, 2010 †: JU (Quebrada Mala Noche)

*chimorum* R. Bohart & Stange, 1965: HU (Pachitea), LO (Yurimaguas)

*cylindricus* Fox, 1899: CU (Sahuaycu), HU (Pachitea), JU (Chanchamayo)

*fritzi* Stange, 1978: LL (Laredo, Samne, Simbal)

*fuscus* (Perty, 1833): HU (Llullapichis, Monzón), LI (Chancay), LL (Simbal), LO (La Chorrera)

*garciai* R. Bohart & Stange, 1965 Endemic: AY (Cangallo)

*harlequinus* R. Bohart & Stange, 1965 Endemic: JU (Huacapistana)

*huascari* R. Bohart & Stange, 1965 Endemic: PA (Pozuzo)

*inca* Kirsch, 1878: JU (Chanchamayo, Huacapistana)

*neffi* Stange, 1978 Endemic: LA (Lambayeque)

*peruvianus* Zavattari, 1912 Endemic: CU (Vilcanota)

*roridus* Zavattari, 1912: LO (Pebas)

*rossi* Bohart & Stange, 1965 Endemic: HU (Monzón)

*scandens* Zavattari, 1913 Endemic: HU (Pachitea)

*shannoni* R. Bohart & Stange, 1965 Endemic: LO (Iquitos)

*sichelianus* (de Saussure, 1855): LA (Lambayeque)

*simulans* R. Bohart & Stange, 1965 Endemic: CA (Jaén)

*smithii* de Saussure, 1855: CU (Vilcanota), HU (Llullapichis, Monzón), SM

*weyrauchi* R. Bohart & Stange, 1965 Endemic: AN (Huaráz)

Subgenus: *Zethusculus* de Saussure, 1855

*brasiliensis* subsp. *fuscatus* R. Bohart & Stange, 1965: CU (Cusco), HU (Tingo María), JU (La Merced), LO (Iquitos, Putumayo)

*carbonarius* F. Smith, 1857: HU (Tingo María)

*imperfectus* Fox, 1899: HU (Tingo María), LO (Pebas)

*magnus* de Saussure, 1852: LO (Iquitos)

*mexicanus* subsp. *lugubris* Perty, 1833: CU, LO

*mexicanus* subsp. *mexicanus* (Linnaeus, 1758): LO

*romandinus* de Saussure, 1852: HU (Llullapichis), UC (Pucallpa)

*westwoodi* de Saussure, 1852: CA (Jaén), LA (Chongoyape, Lambayeque), LI (Chancay), LL (Simbal), PI (Talara)

**Polistinae**

**Epiponini**

***Agelaia* Lepeletier, 1836**

*angulata* subsp. *angulata* (Fabricius, 1804): CU, HU (Llullapichis), JU (La Merced), LO

*cajennensis* (Fabricius, 1798): CU, HU (Llullapichis, Tingo María), JU (Chanchamayo), LO

*centralis* Cameron, 1907: HU (Llullapichis, Tingo María), JU (Chanchamayo), LO, PA, SM, UC

*cornelliana* subsp. *cornelliana* (Richards, 1943): JU (Huacapistana, Oreja de Capelo)

*cornelliana* subsp. *subterranea* Richards, 1978: CU (Cosñipata, Machu Picchu), HU (Sinchona), PA (Oxapampa)

*flavipennis* (Ducke, 1905): HU (Llullapichis), JU, LO, MD, SM

*fulvofasciata* (DeGeer, 1773): CU, HU (Llullapichis), JU (Chanchamayo), LO, SM

*hamiltoni* Richards, 1978: JU (Chanchamayo), UC (Pucallpa)

*lobipleura* subsp. *lobipleura* Richards, 1978: CU (Paucartambo), HU (Tingo María), JU (Chanchamayo), PA (Oxapampa), UC (B. Abad)

*lobipleura* subsp. *melanogaster* Richards, 1978 Endemic: HU (Previsto)

*multipicta* (Haliday, 1836): HU, LO

*myrmecophila* (Ducke, 1905): HU (Tingo María, Llullapichis†), LO†

*nigrescens* Cooper, 2001: JU (San Ramón)

*ornata* (Ducke, 1905): CU, HU (Llullapichis), JU (San Luis de Shuaro), LO

*pallidiventris* (Richards, 1978): HU, JU, PA

*pallipes* subsp. *festae* (Zavattari, 1906): AM, CA, CU, HU (Tingo María), JU (La Merced, Oreja de Capelo), LO, PI, SM, UC (Pucallpa)

*pallipes* subsp. *pallipes* (Olivier, 1791): LO

*panamaensis* (Cameron, 1906): CU, HU

*testacea* (Fabricius, 1804): HU (Llullapichis), JU (Chanchamayo), LO, SM

***Angiopolybia* Araujo, 1946**

*pallens* (Lepeletier, 1836): CU, HU (Llullapichis, Tingo María, Yurac), JU (Chanchamayo), PA (Oxapampa), SM

*paraensis* (Spinola, 1851): CU, HU (Yurac), JU (Perené, San Luis de Shuaro), LO, MD (Avispas), PA

*zischkai* Richards, 1978: CU (Marcapata), HU (Divisoria), JU (Chanchamayo), PA (Oxapampa), UC (Pucallpa)

***Apoica* Lepeletier, 1836**

Subgenus: *Apoica* Lepeletier, 1836

*albimacula* (Fabricius, 1804): AM (Pomacochas), CU (Cosñipata)

*flavissima* van der Vecht, 1973: CU, HU (Llullapichis, Tingo María), LO, MD (Puerto Maldonado), UC (Pucallpa)

*gelida* van der Vecht, 1972: CU, HU (Tingo María), JU, LO

*pallens* (Fabricius, 1804): CU (Urubamba), HU, JU (San Luis de Shuaro), LO

*pallida* (Olivier, 1791): HU, JU (Satipo), LO, PA (Oxapampa), UC (Yarinacocha)

*strigata* Richards, 1978: CU (Cosñipata, Santa Ana), HU (Llullapichis, Monzón, Pachitea, Tingo María), JU (Chanchamayo, Oreja de Capelo, San Ramón), LO (Iquitos), UC (Pucallpa)

*thoracica* du Buysson, 1906: CU, HU (Chinchao, Llullapichis), JU (Chanchamayo), LO, PA (Oxapampa), SM, UC

Subgenus: *Deuterapoica* Dalla Torre, 1904

*arborea* de Saussure, 1854: CU, HU (Monzón), JU (San Ramón), LO

***Asteleoeca* Raw, 1985**

*traili* (Cameron, 1906): LO

*ujhelyii* (Ducke, 1909): HU (Pachitea, Tingo María) , LO (Rio Sucusari)

***Brachygastra* Perty, 1833**

*albula* Richards, 1978†: CU (Cusco, Cadena).

*augusti* (de Saussure, 1854): AY (Luisiana), CA (Jaén), HU (Divisoria, Llullapichis, Pachitea, Tingo María, Yurac), JU (La Merced, San Ramón), LO (Iquitos, Nuevo Requena, Estiron†), PA (Iscozacín), PI (Suyo, Zapayal), SM, UC (Neshuya, Cordillera Azul†, Pucallpa†); CU† (Cadena), MD† (Res. Nac. Tambopata)

*baccalaurea* (R. von Ihering, 1903): CU (Machu Picchu†), JU (Huacapistana, Oreja de Capelo), PI† (Huacabamba)

*bilineolata* Spinola, 1841: CA (San Ignacio), LO (Iquitos†), PA (Dos de Mayo, Pichis, Cerro de Pasco†), CU† (Cadena)

*buyssoni* (Ducke, 1905): HU (Tingo María), LO (Iquitos, Pebas)

*fistulosa* Naumann, 1968

*lecheguana* (Latreille, 1824): CA (Quebrada Nacho†), LA (Motupe, Pacasmayo, Jequetepeque†), LL (San Pedro de Lloc, Pascamayo†), PI (Amotape, Morropón, Suyo), TU (Tumbes), UC (Lake Yarinacocha†)

*moebiana* (de Saussure, 1867): JU (Chanchamayo), LO (Iquitos)

*propodealis* Bequaert, 1943: HU (Tingo María), LO, PA (Iscozacín), UC (Pucallpa), MD† (Puerto Maldonado), CU† (Cadena)

*scutellaris* (Fabricius, 1804): CU (Cosñipata, Marcapata, Santa Isabel, Loromayo†), HU (Tingo María, Panguana†), JU (La Merced, Perené, Satipo), LO (Iquitos, Pebas), PA (Pichis), SM, PU†

*smithii* (de Saussure, 1854): LO (Putumayo, Galicia†), LA† (Jequetepeque), LL† (Pascamayo)

***Chartergellus* Bequaert, 1938**

*amazonicus* Richards, 1978

*nigerrimus* Richards, 1978: no locality given

*punctatior* Richards, 1978: HU (Cayumba, Tingo María), JU, PA (Oxapampa)

*zonatus* Spinola, 1851: LO (Iquitos)

***Charterginus* Fox, 1898**

*fulvus* Fox, 1898: AP, CU, HU (Tingo María†), LO (Yurimaguas, Mishuyacu†), SM, UC (Pucallpa)

*weyrauchi* Richards, 1978 Endemic: HU (Sinchona)

***Chartergus* Lepeletier, 1836**

*artifex* (Christ, 1791): HU (Llullapichis), LO

***Clypearia* de Saussure, 1854**

*apicipennis* (Spinola, 1851): HU (Pachitea)

*duckei* Richards, 1978: HU

*sulcata* (de Saussure, 1854): LO (Amazonas-Napo)

*weyrauchi* Richards, 1978: HU (Tingo María), UC (B. Abad)

***Epipona* Latreille, 1802 [Cooper 2002]**

*media* Cooper, 2002: CU (Cosñipata), HU (Tingo María), JU (Chanchamayo), MD (Avispas), UC (Pucallpa)

*niger* (Brèthes, 1926): HU

*tatua* (Cuvier, 1797): CU, HU (Llullapichis, Tingo María), LO, SM

***Leipomeles* Möbius, 1856**

*dorsata* (Fabricius, 1804): CU, HU, JU (Satipo), LO, MD

*pusilla* (Ducke, 1904)

*spilogastra* (Cameron, 1912): HU (Pachitea), JU, UC

***Metapolybia* Ducke, 1905**

*acincta* Richards, 1978: HU (Llullapichis, Tingo María), UC (Pucallpa)

*aztecoides* Richards, 1978: JU (Satipo)

*cingulata* (Fabricius, 1804): CU, HU, JU (Huacapistana, La Merced, Mariposa, Satipo), SM, UC

*decorata* (Gribodo, 1896): LO, UC

*docilis* Richards, 1978: HU (Divisoria, Pachitea), JU (Huacapistana, Mariposa, San Luis de Shuaro), PA (Oxapampa)

*suffusa* (Fox, 1898): HU (Pachitea, Tingo María), JU (San Luis de Shuaro)

*unilineata* (R. von Ihering, 1904): HU (Pachitea)

***Parachartergus* R. von Ihering, 1904**

*amazonensis* Ducke, 1905: LO (La Sombra), UC (Atalaya, Pucallpa)

*colobopterus* (Lichtenstein, 1796): PI

*flavofasciatus* (Cameron, 1906): PA (Puerto Bermúdez), UC (Atalaya)

*fraternus* (Gribodo, 1892): HU (Llullapichis), JU (Chanchamayo, San Ramón), LO

*fulgidipennis* (de Saussure, 1854): CU (Cosñipata)

*griseus* (Fox, 1898): LO (La Sombra)

*smithii* (de Saussure, 1854): HU (Pachitea, Tingo María), JU (Chanchamayo), PI (Morropón, Suyo), SM, UC (Pucallpa)

*wagneri* du Buysson, 1904: LO (La Sombra)

*weyrauchi* Willink, 1959: CU, HU (Divisoria), JU, LO, PA (Oxapampa)

***Polybia* Lepeletier, 1836**

Subgenus: *Alpha* de Saussure, 1854

*bifasciata* de Saussure, 1854: CU, HU (Yurac), JU (Chanchamayo†), SM, UCc (Pucallpa), LO† (Boqueron Abad)

*quadricincta* de Saussure, 1854: HU (Tingo María), JU (Chanchamayo, La Merced, Oreja de Capelo, San Ramón), LO, SM, UC† (Pucallpa)

*signata* Ducke, 1905: LO (Iquitos)

Subgenus: *Apopolybia* Richards, 1978

*jurinei* de Saussure, 1854: CU, HU (Llullapichis, Tingo María), JU (Chanchamayo), LO (Iquitos†), MD, SM, UC† (Pucallpa, Previsto)

Subgenus: *Cylindroeca* Richards, 1978

*dimidiata* (Olivier, 1791): AP, HU (Llullapichis, Tingo María), JU (Satipo, Chanchamayo†), SM, UC† (Previsto, Pucallpa)

Subgenus: *Formicicola* Richards, 1978

*rejecta* (Fabricius, 1798): CU (Huadquiña, Santa Ana), HU (Llullapichis, Tingo María, Yanayacu†), JU (Chanchamayo, Perené), LO, SM, UC† (Previsto, Pucallpa)

Subgenus: *Furnariana* Richards, 1978

*furnaria* R. von Ihering, 1904: HU (Pachitea)

*richardsi* Cooper, 1993: LO (Nanay)

Subgenus: *Myrapetra* White, 1841

*aequatorialis* subsp. *aequatorialis* Zavattari, 1906: HU (Carpish)

*belemensis* subsp. *belemensis* Richards, 1970: SM

*bistriata* (Fabricius, 1804): AP, CU, HU (Tingo María), JU (Chanchamayo), LO, UC† (Pucallpa)

*catillifex* Möbius, 1856: CU (Marcapata), HU (Tingo María), JU (Chanchamayo, San Ramón), LO (Boqueron Abad†), SM, UC† (Previsto, Atalaya)

*diguetana* du Buysson, 1905: CU, HU (Llullapichis), LO, PA (Oxapampa†), SM, UC† (Pucallpa)

*dimorpha* Richards, 1978: CU (Cosñipata)

*divisoria* Richards, 1978: HU (Divisoria, Sinchona)

*fastidiosuscula* de Saussure, 1854: CA, HU, JU (Chanchamayo†), LA (Chiclayo†), LL, PI, SM (La Masa), TU

*ficaria* Richards, 1978 Endemic: CA (Celendin)

*flavifrons* subsp. *barbatula* Richards, 1978: CA, HU, LA (Guadelupe, Olmos), LL (Chicama, San Pedro de Lloc, Trujillo), PI (Morropón, Piura, Suyo, Zapayal), TU (Pocitos, Tumbes)

*flavifrons* subsp. *hecuba* Richards, 1951: CU (Machu Picchu)

*juruana* R. von Ihering, 1904: AP, CU (Quillabamba, Santa Ana), JU (Chanchamayo, La Merced), PA, UC (Pucallpa)

occidentalis subsp. *bohemani* Holmgren, 1868): HU, UC

*occidentalis* subsp. *occidentalis* (Olivier, 1791): HU (Sinchona), LO, SM, UC (Pucallpa)

*occidentalis* subsp. *venezuelana* Giordani Soika, 1965: TU (Estero Bendito)

*parvulina* Richards, 1970†: UC (Previsto), JU (Chanchamayo)

*platycephala* subsp. *platycephala* Richards, 1951: HU, LO

*platycephala* subsp. *sylvestris* Richards, 1978: JU, UC (B. Abad, Pucallpa)

*scrobalis* subsp. *pronotalis* Richards, 1978

*scrobalis* subsp. *scrobalis* Richards, 1970: CU, HU, LO

Subgenus: *Pedothoeca* Richards, 1978

*brunnea* (Curtis, 1844): JU

*emaciata* Lucas, 1879: CU (Santa Ana), HU (Llullapichis, Pachitea, Tingo María), JU (Chanchamayo, La Merced, Satipo), LO (Nuevo Requena), SM, UC (Neshuya, Pucallpa)

*singularis* Ducke, 1905: HU (Pachitea), LO (Nuevo Requena), UC (Neshuya, Pucallpa)

Subgenus: *Platypolybia* Richards, 1978

*incerta* Ducke, 1907: AY (San Miguel), HU (Previsto, Sinchona, Tingo María†), JU (Satipo, Chanchamayo†), PA (Oxapampa), UC† (San Alejandro)

*procellosa* subsp. *dubitata* Ducke, 1910: CU, HU, JU (Satipo), LO (Boqueron Abad†), UC† (Previsto)

*procellosa* subsp. *procellosa* Zavattari, 1906: JU (Perené), PA (Pichis)

Subgenus: *Polybia* Lepeletier, 1836

*liliacea* (Fabricius, 1804): CU, HU (Llullapichis), LO

*striata* (Fabricius, 1787): AP, CU (Urubamba), HU (Llullapichis, Tingo María†), JU (La Merced, Oreja de Capelo), LO, MD, PA (Oxapampa), SM, UC† (Pucallpa)

Subgenus: *Synoecoides* Ducke, 1905

*depressa* Ducke, 1905: UC (Pucallpa)

Subgenus: *Trichinothorax* Carpenter & Day, 1988

*batesi* Richards, 1978: PA (Puerto Bermúdez)

*eberhardae* Cooper, 1993: HU (Previsto), JU (Chanchamayo), UC (B. Abad, Pucallpa)

*flavitincta* Fox, 1898: CU

*gorytoides* subsp. *sculpturata* Ducke, 1904: LO

*ignobilis* (Haliday, 1836): AM, CA, CU (Machu Picchu), JU (Satipo), LA (Chiclayo, Motupe), LO, PI (Morropón, Piura), PU, SM (Tarapoto), TU (Tumbes)

*micans* Ducke, 1904: HU (Llullapichis, Previsto, Sinchona), JU (Chanchamayo, Perené), PA (Pichis, Puerto Bermudez†), UC (B. Abad, Pucallpa)

*rufitarsis* subsp. *peruviana* Bequaert, 1943: CU, HU (Previsto, Tingo María), JU (Satipo), LO (Boqueron Abad†, Iquitos†), PA (Oxapampa)

*sericea* (Olivier, 1791): CA (Jaén†), CU (Santa Ana)

*tinctipennis* subsp. *tinctipennis* Fox, 1898: HU (Llullapichis), JU (San Ramón), LO, SM, UC† (Pucallpa)

*velutina* Ducke, 1905: CU, HU (Tingo María) †, JU (Chanchamayo), LO (Boqueron Abad†), SM, UC (Pucallpa, Previsto†, Atalaya†)

***Protopolybia* Ducke, 1905**

*acutiscutis* (Cameron, 1907): HU, JU (Chanchamayo), PA (Oxapampa), LO (Iquitos†)

*amarella* Bequaert, 1944: HU (Llullapichis, Pachitea, Tingo María), UC (Pucallpa)

*bella* (R. von Ihering, 1903): HU (Tingo María†), JU (San Ramón)

*biguttata* Bequaert, 1944 Endemic: JU (Chanchamayo, Perené)

*bituberculata* Silveira & Carpenter, 1995: LO (Iquitos, Sucusari)

*chanchamayensis* Bequaert, 1944 Endemic: HU, JU (Chanchamayo, Perené, San Ramón), SM

*chartergoides* subsp. *boshelli* Bequaert, 1938: HU (Iparía, Llullapichis, Tingo María), JU, UC (Pucallpa), CU† (Paucartambo)

*chartergoides* subsp. *chartergoides* (Gribodo, 1891): CU, HU (Llullapichis, Tingo María), JU (Chanchamayo), LO, UC† (Pucallpa)

*emortualis* (de Saussure, 1855): HU (Llullapichis, Tingo María), LO

*exigua* subsp. *binominata* (Schulz, 1906): CU (Cosñipata), HU (Pachitea, Tingo María), JU (Satipo)

*exigua* subsp. *exigua* (de Saussure, 1854): HU (Iparía), JU (La Merced), LO (Iquitos)

*fuscatus* (Fox, 1898): AP, HU, JU (Perené ), LO

*iheringi* Ducke, 1910 Endemic: AP, CU (Marcapata)

*minutissima* (Spinola, 1851): HU, JU (Chanchamayo), LO

*nitida* (Ducke, 1904): HU (Pachitea)

*rubrithorax* Bequaert, 1944 Endemic: HU (Tingo María)

*sedula* (de Saussure, 1854): HU (Llullapichis, Tingo María), JU, LO, PA (Oxapampa), UC† (Pucallpa)

*weyrauchi* Bequaert, 1944 Endemic: JU (San Ramón)

***Pseudopolybia* de Saussure, 1863 [Andena *et al.* 2007]**

*compressa* (de Saussure, 1854): CU, HU, JU (Perené, San Ramón), LO (La Chorrera), MD (Tambopata)

*difficilis* (Ducke, 1905): LO (La Chorrera, Putumayo)

*vespiceps* (de Saussure, 1864): JU, LO, PA (Oxapampa)

***Synoeca* de Saussure, 1852**

*chalibea* de Saussure, 1852: AP, CU (Vilcanota), HU (Llullapichis, Pachitea, Tingo María), JU (Satipo), LO, PA

*septentrionalis* Richards, 1978: PI (Suyo, Zapayal), TU (La Palma, Papayal, Pocitos, Tumbes), (“Las Virgenes”, “Tambillo”)

*surinama* (Linnaeus, 1767): HU (Llullapichis, Tingo María), JU (Chanchamayo), LO, UC (Pucallpa)

*virginea* (Fabricius, 1804): CU, HU (Llullapichis, Tingo María)

**Mischocyttarini**

***Mischocyttarus* de Saussure, 1853**

Subgenus: *Clypeopolybia* Brèthes, 1923

*carbonarius* subsp. *carbonarius* (de Saussure, 1854): HU (Llullapichis), LO

*flavicans* subsp. *flavicans* (Fabricius, 1804): AM, HU (Iparía), LO

*woytkowskyi* Richards, 1978 Endemic: CU (Cosñipata)

Subgenus: *Haplometrobius* Richards, 1978

*chanchamayoensis* Richards, 1978: JU (Chanchamayo, San Ramón), PA (Pozuzo)

*decimus* Richards, 1978

*dimorphus* Zikán, 1949 Endemic: JU (Chanchamayo)

*elegantulus* Zikán, 1949: HU (Tingo María)

*filipendulus* Cooper, 1998: LO (Nanay)

*illusorius* Richards, 1978: CU (Santa Ana)

*interruptus* Richards, 1978: LO (Nanay)

*longicornis* Zikán, 1949 Endemic: PA (Oxapampa)

*melanoleucus* Richards, 1978 Endemic: HU (Previsto), UC (Pucallpa)

*melanoxanthus* Richards, 1978

*minifoveatus* Cooper, 1998 Endemic: JU (San Ramón)

*mifificus* Zikán, 1949

*montivagus* Cooper, 1996: PA (Villa Rica)

*nigropygialis* Zikán, 1949 Endemic: JU (Oreja de Capelo), PA (Oxapampa)

*ornatus* Zikán, 1949: JU (Oreja de Capelo, San Ramón)

*peruanus* Zikán, 1949 Endemic: CU (Santa Ana)

*peruviensis* Richards, 1945: JU (Perené), PA (Pozuzo)

*reflexicollis* Zikán, 1949: HU (Tingo María)

*silvicola* Zikán, 1949: HU (Previsto), JU (San Ramón)

*surinamensis* subsp. *surinamensis* (de Saussure, 1854): JU

*synoecus* Richards, 1940: LO

*tectus* Cooper, 1996: HU (Tingo María)

*tenuis* Richards, 1945 Endemic: CU, HU (Tingo María)

*weyrauchi* Zikán, 1949: HU (Tingo María), JU (San Luis de Shuaro)

*xanthocerus* Richards, 1945: HU (Tingo María)

Subgenus: *Kappa* de Saussure, 1853

*imitator* (Ducke, 1904): HU (Tingo María), JU (Chanchamayo, Perené), LO (Tapiche), MD (Cocha Cashu), UC (Acobamba)

*injucundus* (de Saussure, 1854): CU?, HU (Previsto, Tingo María), JU (Satipo), LO (Iquitos, Rio Sucusari), UC (Pucallpa)

*latissimus* Richards, 1978: LO (Iquitos)

*metathoracicus* (de Saussure, 1854): LO (Iquitos, Tapiche)

*mysticus* Oliveira, 2006 Endemic: no locality given

*paris* Silveira, 2006 Endemic: MD (Avispas)

*pseudomimeticus* (Schulz, 1904): HU (Llullapichis), LO (Iquitos)

*schunkei* Zikán, 1949 Endemic: JU (Chanchamayo)

Subgenus: *Megacanthopus* Ducke, 1904

*malaris* Richards, 1978: LO (Cerro Azul, Contamana)

*melanopygus* Richards, 1945: JU (La Merced, San Luis de Shuaro, San Ramón, Satipo), LO ( Iquitos)

*saturatus* Zikán, 1949: HU (Tingo María), JU (La Merced)

Subgenus: *Mischocyttarus* de Saussure, 1853

*cinerasceus* Zikán, 1949 Endemic: JU (Oreja de Capelo), PA (Oxapampa)

*drewseni* subsp. *andinus* Zikán, 1949: AP (Abancay), AY (Ayacucho, Huanta, Ninabamba, San Miguel), CA, CU (Urubamba), HU (Huánuco), HV (Anco), JU

*drewseni* subsp. *drewseni* de Saussure, 1857: AY, CU, LO

*gynandromorphus* Richards, 1945: JU (Perené), PA (Pichis)

*labiatus* (Fabricius, 1804): AM, HU (Llullapichis), HV, JU, LO, MD

*mattogrossoensis* Zikán, 1935: CU

*rotundicollis* (Cameron, 1912): AP (Abancay), AY (Luisiana, Ninabamba, Pampas, San Miguel, San Francisco, Sivia), CU, HU (Huánuco, Pachitea), JU (Chanchamayo, San Ramón, Satipo), PA, UC (Neshuya)

*smithii* de Saussure, 1853: LO

*tomentosus* Zikán, 1935: AM, HU (Llullapichis), JU (Chanchamayo, La Merced, San Luis de Shuaro, Satipo), LO

Subgenus: *Monogynoecus* Richards, 1941

*foveatus* Richards, 1941: UC (B. Abad)

*insolitus* Zikán, 1949: HU (Carpish)

*lecointei* Ducke, 1904

Subgenus: *Omega* de Saussure, 1854

*vaqueroi* Zikán, 1949: HU (Tingo María), PA (Pozuzo)

Subgenus: *Phi* de Saussure, 1854

*alfkenii* Ducke, 1904†: LO

*barbatulus* Richards, 1978: HU (Chinchao)

*commixtus* Richards, 1978 Endemic: JU (Oreja de Capelo), PA (Dos de Mayo, Pichis)

*flavicornis* Zikán, 1949: CU (Santa Ana, Cusco†, Urubamba†), HU (Tingo María, Huánuco†), JU (La Merced, San Ramón, Satipo), LO, PA (Oxapampa) (=*flavicornis* subsp. *nigricornis* Zikán, 1949, see in Silveira (2013))

*flavoniger* Zikán, 1949: JU (Oreja de Capelo, San Ramón, Valle Chanchamayo†)

*hirtulus* Zikán, 1949 Endemic: JU (Huacapistana)

*imeldai* Zikán, 1949: JU (Chanchamayo)

*inca* Zikán, 1949 Endemic: no locality given

*oreophilus* Zikán, 1949: CU (Machu Picchu)

*peduncularius* Zikán, 1949 Endemic: JU (Chanchamayo)

*petiolatus* Zikán, 1949: CA (Jaén, San Ignacio), PI (Piura)

*piceus* Zikán, 1949 Endemic: JU (Chanchamayo), PA (Oxapampa)

*rufipes* Zikán, 1949 Endemic: JU (Huacapistana)

*tarmensis* Richards, 1945 Endemic: JU (Huacapistana, Oreja de Capelo)

*tayacaja* Silveira, 2013† Endemic: HV (Campo Armiño)

*transandinus* Richards, 1978 Endemic: LA (Olmos)

Subgenus: *Scytokeraia* Cooper, 1997

*moralesi* Zikán, 1949: JU (Oreja de Capelo, San Ramón)

*rhadinomerus* Cooper, 1997 Endemic: AM (Rodríguez de Mendoza)

*subornatus* Zikán, 1949 Endemic: JU (San Ramon), PA (Oxapampa, Villa Rica)

**Polistini**

***Polistes* Latreille, 1802**

Subgenus: *Aphanilopterus* Meunier, 1888

*adelphus* Richards, 1978: TU (Tumbes)

*aterrimus* de Saussure, 1853: AM (Pomacochas), AR, CU (Machu Picchu), HU (Chinchao), JU (Huacapistana, La Merced, Oreja de Capelo, Pichita, Tarma, San Ramón, ), PA (Oxapampa)

*bicolor* Lepeletier, 1836: CU, HU (Llullapichis), JU (Chanchamayo, Perené), LO (Iquitos), SM

*billardieri* subsp. *biglumoides* Ducke, 1904: AY (Ninabamba), CU, HU (Huánuco)

*canadensis* subsp. *canadensis* (Linnaeus, 1758): CA (Celendin), CU (Urubamba), HU (Ambo, Huánuco, Tingo María), JU (Satipo)

*carnifex* subsp. *boliviensis* Bequaert, 1936: JU (Chanchamayo, Perené)

*carnifex* subsp. carnifex (Fabricius, 1775): LO, SM, UC

*claripennis* Ducke, 1904: LO (Iquitos), UC (B. Abad, Pucallpa†)

*deceptor* Schulz, 1905: HU (Sinchona, Jumbatis), JU (La Merced, Perené, San Luis de Shuaro, San Ramón, Satipo, Chanchamayo†), LI (Chosica), MD (Iberia), UC† (Pucallpa), SM† (Rioja)

*erythrocephalus* Latreille, 1813: HU, MD

*goeldii* Ducke, 1904: HU (Llullapichis, Tingo María), JU (Chanchamayo, Satipo), LO

*huacapistana* Richards, 1978 Endemic: JU (Huacapistana)

*infuscatus* subsp. *anduzei* Bequaert, 1943: CU (Callanga)

*infuscatus* subsp. *infuscatus* Lepeletier, 1836: AM, JU (La Merced)

*lanio* subsp. *lanio* (Fabricius, 1775): CA, CU, HU (Llullapichis), JU, LO

*major* subsp. *weyrauchi* Bequaert, 1936: CA, HU (Huánuco), JU (Oreja de Capelo, San Ramón), LO

*maranonensis* Willink, 1964: CA (Bellavista, Cajamarca, Celendin, Chontabamba, Chusgón, Jaén, Lajas, Limón, San Ignacio, Sunchubamba), AM† (Valley of Rio Utcubamba)

*ninabamba* Richards, 1978 Endemic: AY (Ninabamba)

*occipitalis* Ducke, 1904: HU, JU (Satipo), LO (Iquitos)

*pacificus* Fabricius, 1804: HU (Tingo María), JU (Satipo), LO (La Chorrera, Putumayo), PA (Pozuzo), UC (Pucallpa)

*pacificus* subsp. *modestus* Smith, 1868†: HU (Tingo María)

*peruvianus* Bequaert, 1934 Endemic: AN, AR (Arequipa), IC (Chincha, Palpa), LA (Chiclayo, Pacasmayo), LI (Cañete, Chosica, Huaura, San Bartolomé, Verrugas), LL (San Pedro de Lloc, Trujillo), PI

*rufiventris* Ducke, 1904: LO

*testaceicolor* Bequaert, 1937: HU, JU (La Merced, San Ramón), LO (Pucallpa†), UC† (Previsto)

*versicolor* subsp. *flavoguttatus* Bequaert, 1934: CU (Santa Ana, Mándor), MD

*versicolor* subsp. *versicolor* (Olivier, 1791): AP (Abancay, Curahuasi), AY (Huanta, Luisiana, Ninabamba, San Francisco, San Miguel, Sivia, Teresita), CA (Querocotillo), CU (Limatambo, Pichari), HU (Pachitea, Tingo María), HV (Anco, Campo Armiño), JU (Chanchamayo, Pampa Silva, San Ramón, Satipo), LA (Motupe), LO (Nuevo Requena), PA (Oxapampa), PI (Morropón, Piura, Talara, Zapayal), SM (Tarapoto), TU (Tumbes), UC (Cachi, Neshuya, Pucallpa)

*weyrauchorum* Willink, 1964: AN (Cajacay, Yungay), AY (San Pedro), AR (Arequipa, Atiquipa, Camaná, Caravelí, Ocoña), CA (Cascas, Chilete, Limón, Tembladera), IC (Ica, Pisco), LA (Chiclayo, Guadelupe, Motupe), LI (Atocongo, Cañete, Chillón, Chosica, Churín, Cocayalta, Huaura, Huinco, La Molina, Lachay, Lima, Matucana, Q. Verde, Quilmaná, Pachacámac, Pueblo Libre, Verrugas, San Bartolomé, Santa Eulalia, Santa Rosa de Quives, Surco), LL (Cartavio, Chan-chan, Chicama, Pacasmayo, Paiján, San Pedro de Lloc, Trujillo), PI (Paita), TA (Tacna)

*xanthogaster* subsp. *willei* Bequaert, 1940 Endemic: AP (Abancay), AY (Ayacucho, Cangallo, Chacco, Huatatas, Huanta, Ninabamba, Río Blanco, San Miguel), CU (Limatambo), HV (Anco, Colorado, La Mejorada, Mariño, Pachachaca, Santo Tomás).
